# Data-driven distillation and precision prognosis in traumatic brain injury with interpretable machine learning

**DOI:** 10.1038/s41598-023-48054-z

**Published:** 2023-12-01

**Authors:** Andrew Tritt, John K. Yue, Adam R. Ferguson, Abel Torres Espin, Lindsay D. Nelson, Esther L. Yuh, Amy J. Markowitz, Geoffrey T. Manley, Kristofer E. Bouchard, the TRACK-TBI Investigators, C. Dirk Keene, Christopher Madden, Michael McCrea, Randall Merchant, Pratik Mukherjee, Laura B. Ngwenya, Claudia Robertson, David Schnyer, Sabrina R. Taylor, Ross Zafonte

**Affiliations:** 1 Applied Math and Computational Research Division, Lawrence Berkeley National Laboratory, Berkeley, CA USA; 2 Brain and Spinal Injury Center, Zuckerberg San Francisco General Hospital and Trauma Center, San Francisco, CA USA; 3 Department of Neurosurgery, Weill Institute for Neurosciences, University of California San Francisco, San Francisco, CA USA; 4 San Francisco Veterans Affairs Healthcare System, San Francisco, CA USA; 5 Departments of Neurosurgery and Neurology, Medical College of Wisconsin, Milwaukee, WI USA; 6 Weill Neurohub, University of California San Francisco, San Francisco, CA USA; 7 Weill Neurohub, University of California Berkeley, Berkeley, CA USA; 8 Scientific Data Division, Lawrence Berkeley National Laboratory, Berkeley, CA USA; 9 Biological Systems and Engineering Division, Lawrence Berkeley National Laboratory, Berkeley, CA USA; 10 Helen Wills Neuroscience Institute and Redwood Center for Theoretical Neuroscience, University of California Berkeley, Berkeley, CA USA; 11 University of Washington, Seattle, USA; 12 UT Southwestern, Dallas, USA; 13 Medical College of Wisconsin, Milwaukee, USA; 14 Virginia Commonwealth University, Richmond, USA; 15 University of California, San Francisco, San Francisco, USA; 16 University of Cincinnati, Cincinnati, USA; 17 Baylor College of Medicine, Houston, USA; 18 UT Austin, Austin, USA; 19 Harvard Medical School, Boston, USA

**Keywords:** Brain injuries, Machine learning

## Abstract

Traumatic brain injury (TBI) affects how the brain functions in the short and long term. Resulting patient outcomes across physical, cognitive, and psychological domains are complex and often difficult to predict. Major challenges to developing personalized treatment for TBI include distilling large quantities of complex data and increasing the precision with which patient outcome prediction (prognoses) can be rendered. We developed and applied interpretable machine learning methods to TBI patient data. We show that complex data describing TBI patients' intake characteristics and outcome phenotypes can be distilled to smaller sets of clinically interpretable latent factors. We demonstrate that 19 clusters of TBI outcomes can be predicted from intake data, a ~ 6× improvement in precision over clinical standards. Finally, we show that 36% of the outcome variance across patients can be predicted. These results demonstrate the importance of interpretable machine learning applied to deeply characterized patients for data-driven distillation and precision prognosis.

## Introduction

The collection of ever larger and more detailed biomedical datasets brings with it the promise of personalized treatments and interventions for a diversity of diseases and disorders^1^. Extraction of clinically interpretable insights from such large, complex datasets is challenging and creates an impediment to better understanding and hence treatment. Current medical frameworks typically group patients with a given condition into a small number of classes, obfuscating the individual nature of their biology and ailments^2^. A critical first step towards personalized treatments is to increase the precision with which we describe the patient and their outcomes, and predict those outcomes from socioeconomic, demographic, biomarker, and medical variables from initial clinical presentation, that we refer to as “intake” data^2,3^. Here, we addressed this gap by developing and applying interpretable machine learning techniques for data distillation and precision prognoses in the context of traumatic brain injury (TBI).

Traumatic brain injury is damage to the brain resulting from any external force or object. According to 2020 estimates, 2.8 million people sustain a TBI annually in the United States (US), of which 64,000 die, 223,000 are hospitalized, and 2.5 million (~ 90%) are treated and released from an emergency department^4^. TBI is a contributing factor to one-third of all injury-related deaths in the US and has complex relationships with polytrauma^5^. Direct medical costs and indirect costs of TBI, such as lost productivity, cost the world economy ~ $400 billion annually^6^. Despite the tremendous human and financial toll of TBI, our understanding of, and hence ability to treat this disabling medical condition, is not commensurate with its personal and societal impact^6^.

**Figure 1:**
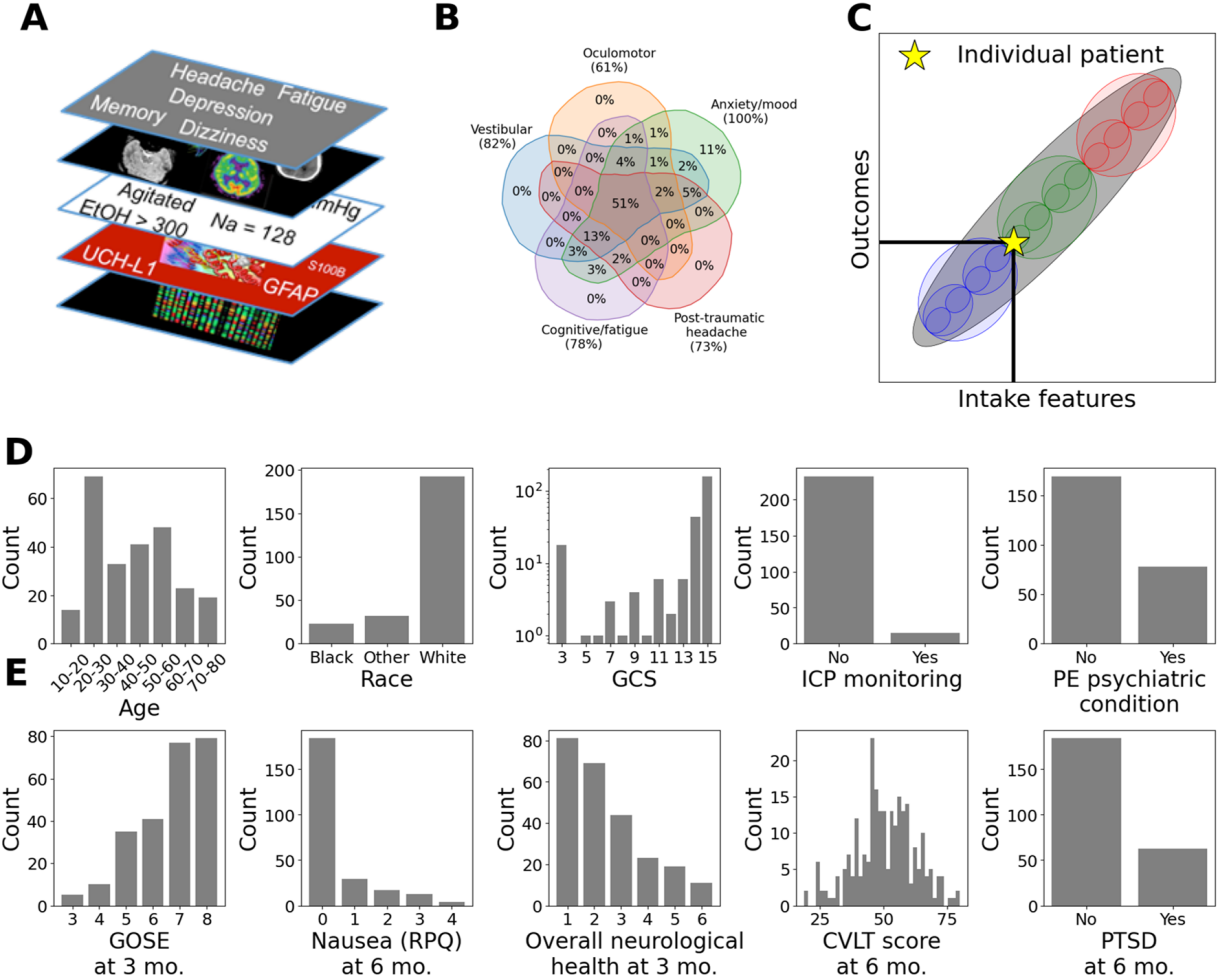
TBI patients and outcomes are described by complex combinations of heterogeneous clinical and outcome features. (**A**) Schematic depicting the diverse variable types across many scales that are collected in the TRACK-TBI Pilot data set. (**B**) Venn diagram of co-morbidity of post-TBI clinical symptoms at 3 months. Outcome variables were put into mutually exclusive groups based on the type of symptom measured by the variable. Percentages depict the fraction of patients that have symptoms associated with these outcome phenotypes (**C**). Precision medicine and personalized treatments require prognostication (i.e., prediction of outcomes) at the individual patient level. Conceptually, broad classifications of patient outcomes (e.g., mild, moderate, severe) can be subdivided into finer categories for more precise patient prognosis. Carrying this subdivision of categories (i.e., colored ovals) forward to the individual patient level turns the classification problem into a regression problem (i.e., the single gray oval across all features). (**D**) Histograms of selected clinical intake features. € Histogram of selected clinical outcome features. Both intake and outcome features contained binary variables (e.g., intracranial pressure monitoring or post-traumatic stress disorder), ordinal variables (e.g., age or overall neurological health), and more continuous variables (e.g., California Verbal Learning Test). *CVLT* California Verbal Learning Test, *EtOH* alcohol, *GCS* Glasgow Coma Scale, *GFAP* glial fibrillary acidic protein, *GOSE* Glasgow Outcome Scale-Extended, *ICP* intracranial pressure, *Na* sodium, *PE* pre-existing, *PTSD* post-traumatic stress disorder, *RPQ* Rivermead Postconcussion Symptom Questionnaire, *UCH-L1* ubiquitin c-terminal hydrolase-L1.

The Transforming Research and Clinical Knowledge in Traumatic Brain Injury Pilot Study (TRACK-TBI Pilot; ClinicalTrials.gov NCT01565551; enrollment 2010-2012^7^) consortium aimed to improve our diagnostic models of TBI, understand patient outcomes, and predict those outcomes from the intake features^8,9^. To this end, the study collected multimodal data immediately following injury (e.g., blood based biomarkers, radiological assessments of CT scans, as well as questions assessing a variety of socio-economic, pre-existing conditions) and diverse outcome measures at 3, 6 and 12 months post injury, to deeply phenotype patients (Fig. 1A). The enrolled patients (a subpopulation of TBI patients who were admitted to the ER/hospital/ICU) were diverse across demographic and socioeconomic characteristics, pre-existing conditions, and injury causes and severity which, together, could lead to complex and diverse outcome phenotypes with numerous comorbidities. Indeed, as qualitatively displayed by the Venn diagram in Fig. 1B, in the TRACK-TBI Pilot data (N = 586 patients), individual patients exhibited complex combinations of multiple clinical symptoms at 3 months.

One contributing factor to the dearth of effective treatments for TBI is the lack of holistic yet precise descriptions of TBI patient characteristics to allow for a more personalized approach to care. Causes of TBI include a range of mechanisms from blunt force trauma to blast injuries. Further exacerbating the complexity of the situation, each TBI patient’s presentation is coupled with distinct sets of comorbidities that may or may not contribute to their specific outcomes^10–14^. For example, as with many diseases and disorders, it is likely that heterogeneity in preexisting medical conditions and socioeconomic status contribute to the diversity of TBI outcomes^10,15, 16^. The raw data describing TBI patients are both high-dimensional and complex, and extracting clinical insights that holistically describe patients' intake characteristics and outcome phenotypes is an open challenge. Currently, standard clinical metrics typically classify TBI as either mild, moderate, or severe^17,18^. As the precision of describing outcomes increases from broad categories towards smaller patient subsets (depicted as smaller ovals in Fig. 1C) all the way to individual patients (demarcated with a star in Fig. 1C), the prediction problem transitions from a classification to a regression problem (Fig. 1C). Predictions of precise yet holistic patient outcome phenotypes from intake characteristics is nascent. From a data analytics perspective, these gaps correspond to three challenges: to distill high-dimensional, complex intake and outcome data in terms of holistic, clinically meaningful concepts; to increase the precision with which TBI outcomes are described; and to determine the degree to which precise outcomes can be predicted from intake data (prognosis).

We hypothesized that, hidden within the complexity of raw TRACK-TBI Pilot data, there were a small number of clinical concepts that parsimoniously described individual patients' intake characteristics and outcome phenotypes. We further hypothesized that there is an unappreciated level of precision with which TBI outcome phenotypes can be predicted from intake features. To test these hypotheses, we developed interpretable statistical-machine learning algorithms and applied them to demographic, socioeconomic, biomarker, and medical record data (‘intake’ features) as well as patient outcome data. We found that a small number of latent factors captured holistic clinical concepts associated with individual patients' intake characteristics and outcome phenotypes. Across the patient population, we found that 19 TBI outcome clusters are predictable from intake data. Furthermore, we found that 36% of the total variance across individual patient outcomes can be predicted. Pre-existing sociomedical factors, mental health conditions, and other medical conditions interacted with injury severity indicators to predict diverse TBI outcomes. Together, these results deepen our understanding of TBI by providing quantitative, data-driven descriptions of holistic TBI patient intake characteristics and outcome phenotypes, and enable state-of-the-art prediction of precise TBI outcomes down to the individual patient level. While we here demonstrate the power of our approach in the specific context of TBI, our conceptual framework and specific methods are generalizable. As such, we believe our work will have applications to other complex medical conditions in the emerging age of data-driven precision medicine.

## Results

### TBI patients are described by complex combinations of heterogeneous intake and outcome features

The TRACK-TBI Pilot data set has been described previously and consists of a broad and detailed set of variables from 586 TBI patients evaluated at three United States Level 1 Trauma Centers^7^ (see “Methods” section). From these patients, there were up to 524 ‘intake’ variables collected in the first 24 h of admissions (i.e., features) describing socioeconomic, demographic (race, sex, etc.,), pre-existing conditions (e.g., depression, cardiovascular, gastrointestinal, etc.,), and clinical intake information (e.g., location of injury, Glasgow Coma Scale (GCS), highest level of care and hospital course, etc.,) (Fig. 1D). For the same patients, there were up to 394 outcome items from each follow-up assessment in the cognitive, psychiatric, functional, return to work, quality of life, and other domains (Fig. 1E). Due to several causes (e.g., death/coma, lack of patient participation in follow-up assessments, etc.), not all variables are present for all patients. Our goal was to determine the degree of precision prognosis that can be achieved in the most deeply phenotyped individuals. Thus, we focused on a subset of patients who were alive and not comatose (e.g., vegetative state) at 3 or 6 months post-injury and for whom there were no missing values in a large subset of intake and outcome features (i.e., a ‘complete analysis’). This sub-setting resulted in *N* = 247 patients, *P*_*I*_ = 235 intake features and *P*_*O*_ = 177 outcome features. There were 174 males and 73 females. Of the 247 patients, 33% were discharged home, 29% were admitted to the ICU, 20% were admitted to the ward, 14% were stepdown, and 4% went into surgery. The 177 outcome features span a diverse range of clinical assessment domains. This included both clinical instruments and individual clinical features. The data types associated with both the intake features and outcome features were heterogeneous and complex. For example, some features were multi-categorical (e.g., race), some were binary (e.g., intracranial pressure monitoring; 6-month post-traumatic stress disorder) or ordinal across different ranges (e.g., GCS; and Glasgow Outcome Scale-Extended: GOSE) (Fig. 1D,E). Subsequently, all data were converted to non-negative numerical values and numerical ranges were standardized (see “Methods” section). Our strategy was to first distill the complexity of the original intake and outcome feature sets by learning interpretable latent factors that enhance clinical insight into individual patients. We then determined the precision by which holistic TBI outcomes can be predicted from intake features, and finally interpreted those predictions through the lens of the learned latent factors.

### Complex TBI patient data can be distilled to clinically interpretable latent factors

The high-dimensional feature sets associated with both the intake and outcome data contain all available information for description of patients’ intake features, injury characteristics, and outcomes. At the surface, it would appear that one benefit of working with the raw features is that, by definition, the item-level description may be clinically interpretable, as they were designed by clinicians. Previous approaches have interrogated the data at the item level^5,8,12^. However, holistically, individual patients are defined by combinations of a large number of features, and the presence of complex and often hidden (i.e., latent) relationships amongst the features^19^. Indeed, principal components analysis (PCA, Supplementary Fig. 1A,B) shows that a small number of principal components retain the majority of the variance in each feature set (3 and 12 PCs account for 95% of variance in intake and outcome features). This indicates that, while there are a large number of (correlated) features, the information they contain can be captured by a much smaller number of latent factors (3/235 = 1.3% and 12/177 = 6.8% for intake and outcome feature sets respectively). However, while PCA is optimal for capturing variance, it is often challenging to interpret individual components in terms of clinically meaningful concepts.

**Figure 2:**
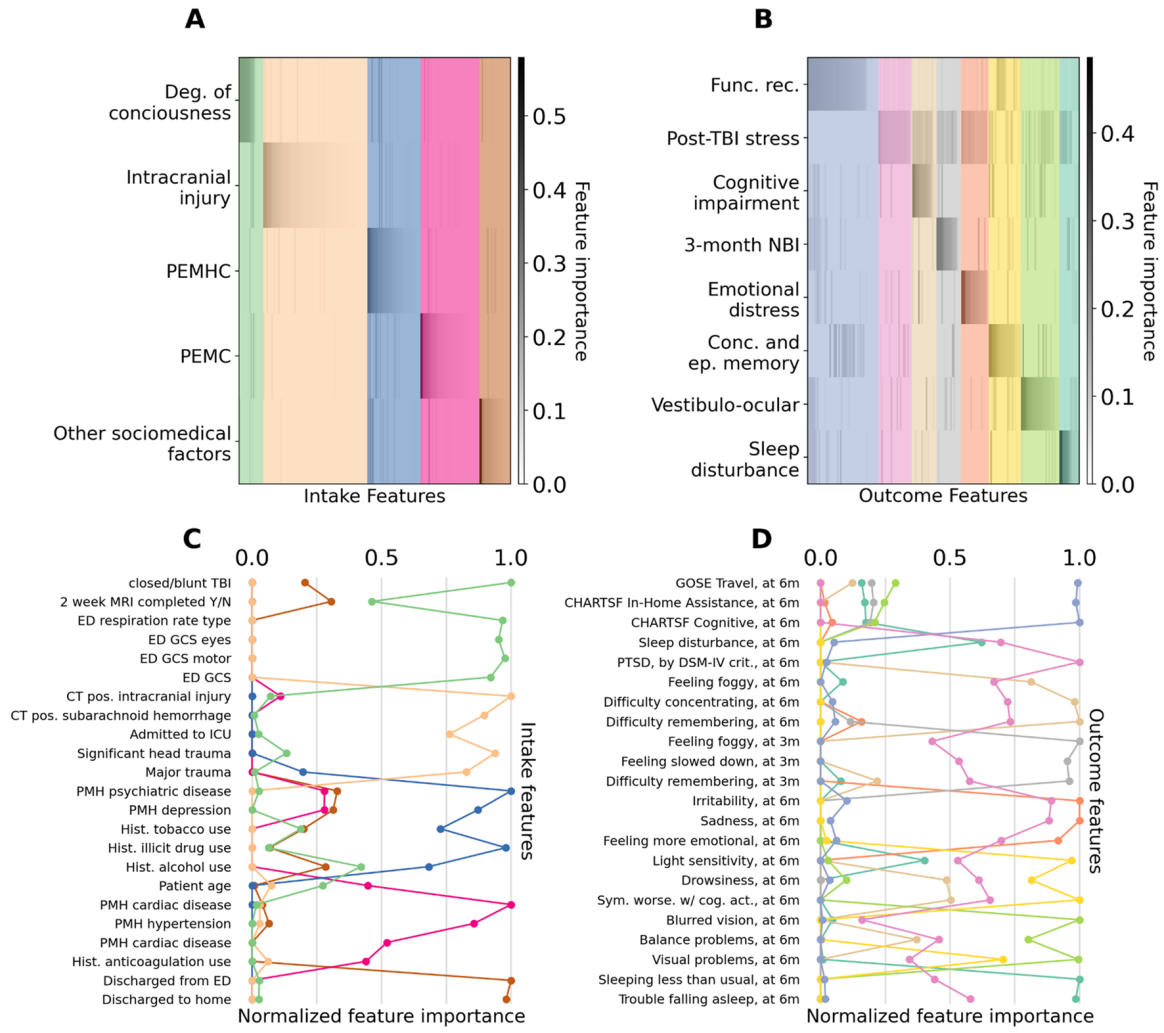
Complex TBI patient data can be distilled to clinically interpretable latent factors. Visualization of the contribution of intake and outcome features to extracted latent factors. (**A**,**B**) Latent factors (rows) and original features (columns) for intake (**A**) and outcome (**B**) features. Results are ordered for visualization purposes. Latent factors are labeled according to the holistic contributions of the individual features to each factor. Columns were shaded to indicate the features that contribute the most to each latent factor. Note that individual intake (**A**) and clinical features (**B**) contributed to a small number of latent factors, and each latent factor was constructed from a small-number of original features (**C**,**D**) Top contributing features for the intake (**C**) and outcome (**D**) latent factors. The normalized weight of the top features for each latent factor is plotted. *3m* 3-month, *6m* 6-month, *CHARTSF* Craig Handicap Assessment and Reporting Technique Short Form, *CT* computed tomography, *DSM-IV* Diagnostic and Statistical Manual of Mental Disorders, 4th Edition, *ED* emergency department, *GCS* Glasgow Coma Scale, *GOSE* Glasgow Outcome Scale-Extended, *NBI* neurobehavioral impairment, *PEMC* pre-existing medical conditions, *PEMHC* pre-existing mental health conditions, *PMH* prior medical history, *PTSD* post-traumatic stress disorder, *TBI* traumatic brain injury. Coloring for latent intake features: light green = degree of consciousness; light orange = severity of intracranial injury; blue = PEMHC; deep pink = PEMC; brown = sociomedical factors. Coloring for latent outcome features: light blue = functional recovery; pink = post-TBI stress; light brown = cognitive impairment; gray = 3-month neurobehavioral impairment; orange = emotional distress, yellow = concussive & episodic memory issues; lime green = vestibulo-ocular symptoms; turquoise = sleep disturbance.

To learn a small number of latent factors that group correlated features into clinically meaningful concepts, we deployed nonnegative matrix factorization (NMF) using our recently introduced UoI-NMF algorithm. UoI-NMF learns stable latent factors of the data that group correlated variables together. UoI-NMF was performed on the intake features and outcome features separately. We found that five (5/235 = 2.1%) and eight (8/177 = 4.5%) latent factors parsimoniously summarized the intake and outcome features, respectively (Supplementary Fig. 1C,D). Generally speaking, we found that a relatively small set of original features were strongly loaded (weighted) on each factor, and that features contributing most to each factor reflect the same or similar conceptual groups (Fig. 2A,B) (see also Supplementary Fig. 2A). Furthermore, individual features that were indicative of the same clinical concept were naturally grouped together (Fig. 2C,D) (See also Supplementary Figs. 3 and 4). These qualities enabled us to assign clinically interpretable labels to the latent factors based on the features that they were composed of.

Figure 2A presents the contributions (gray scale color bar) of individual intake features (x-axis) to the extracted latent factors (y-axis). We found that latent factors grouped the intake features into clinical concepts named as follows (based on the features prominent in those factors): degree of consciousness, severity of intracranial injury, pre-existing mental health conditions, pre-existing medical conditions, and sociomedical factors. The contribution of a subset of the most important features for each intake factor are displayed in Fig. 2C (see Supplementary Fig. 3 for further details). We found that the latent factors grouped conceptually similar intake features together. For example, the degree of consciousness factor heavily weighted features such as closed injury and good responses on the Glasgow Coma Scale (GCS), indicating that patients high in this factor would have a high degree of consciousness. The intracranial injury factor heavily weighted significant trauma and intracranial injury features, indicating that patients high in this factor would have severe intracranial injury. Interestingly, the sociomedical factor was composed of presentation with pre-existing mental health conditions, substance use, injury by assault, and non-white race, but were discharged from the hospital (Fig. 2c, see also Supplementary Fig. 3).

Likewise, for the outcome features, we found that the latent factors holistically captured several clinically meaningful outcome phenotypes. Figure 2B presents the contributions (gray scale color bar) of individual outcome features (x-axis) to the extracted latent factors (y-axis). We found that latent factors grouped the outcome features into the following clinical concepts: functional recovery, post-TBI stress, cognitive impairment, 3-month neurobehavioral impairment, emotional distress, concussive and episodic memory issues, vestibulo-ocular symptoms, and sleep disturbance. The contribution of a subset of the most important features for each factor are displayed in Fig. 2D (See Supplementary Fig. 4 for further details). Interestingly, in contrast to the intake latent factors, the latent factors for outcomes had more overlap in their contributing features (Fig. 2A vs. Fig. 2B). This supports the observation that TBI patients generally exhibit a complex combination of outcome phenotypes^20^. In particular, the post-TBI stress factor was composed of PTSD-specific features (e.g., PTSD diagnosis), but also had contributions from many other features that spanned other outcome phenotypes, such as sleep disturbance, depression, and neuro-behavioral impairments. Together, these observations indicate that the latent factors succinctly and holistically capture complex clinical concepts that underlie the intake characteristics and outcome phenotypes of TBI patients.

### The quantitative phenotypic landscape of individual TBI patients

The results presented above revealed a small number of clinically interpretable latent factors (5 and 8) that parsimoniously summarize the high-dimensional intake and outcome features across patients. These factors not only distill the complexity of the original features, but can be utilized as quantitative descriptors of the individual patients. That is, the full description of each patient in terms of the original features can be reconstructed as additive combinations of the NMF latent factors. This enables, among other things, the quantitative assessment of the presence and degree to which the characteristics and phenotypes (summarized by the intake and outcome latent factors) are expressed by individual patients. Therefore, we next analyzed the landscape of individual TBI patients through the lens of the clinically interpretable intake and outcome latent factors.

**Figure 3:**
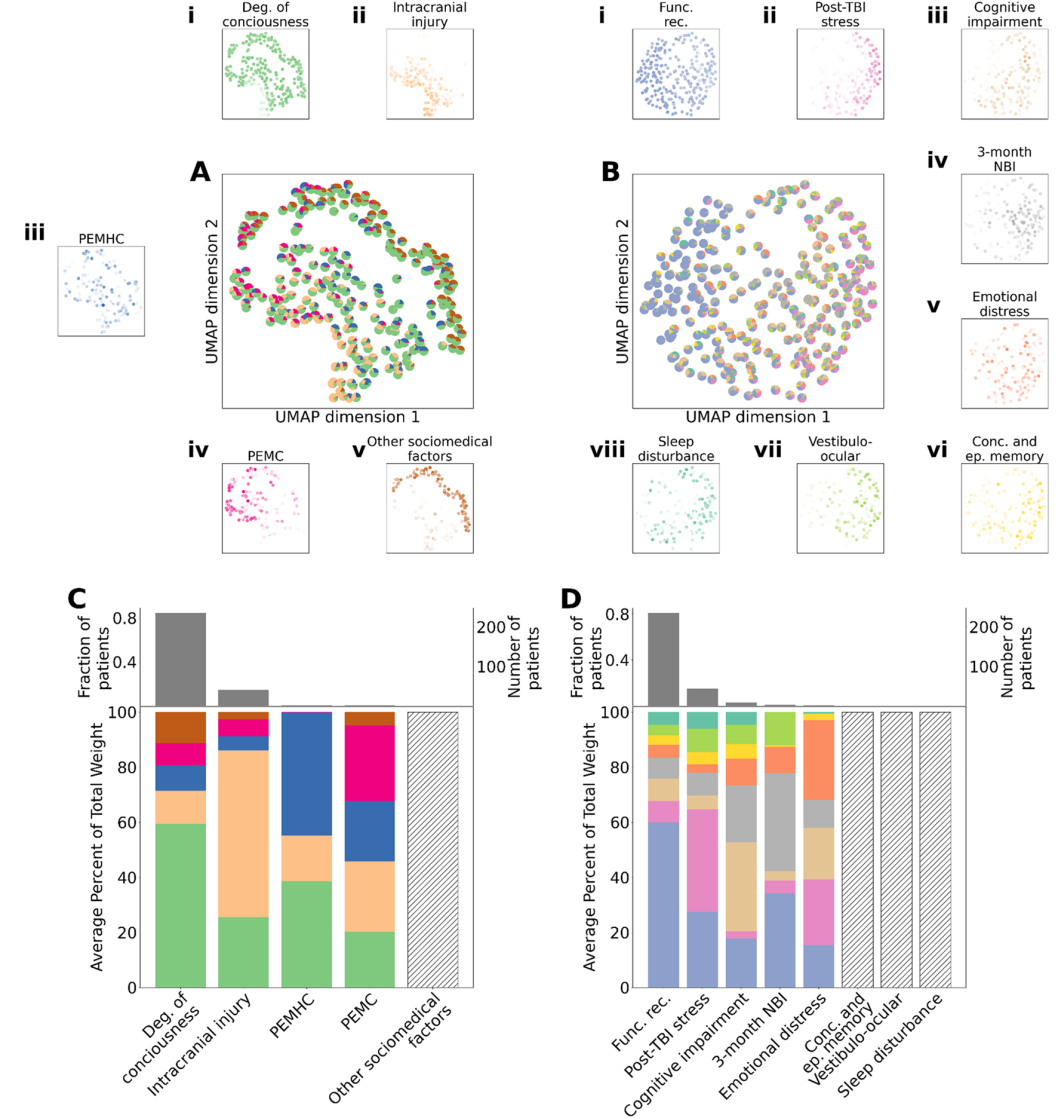
The quantitative phenotypic landscape of individual TBI patients. (**A**) Two-dimensional UMAP embedding of clinical features. Each point (i.e., patient) in the two-dimensional embedding is represented as a pie chart computed using normalized weights from NMF performed on the clinical features. (**B**) Two-dimensional UMAP embedding of outcome features. (**A.i**–**A.v**) Probability densities for clinical feature UMAP embeddings. Points in the kernel density estimates were weighted by the respective clinical feature NMF factor weights. (**B.i**–**B.viii**) Probability densities for outcome feature UMAP embeddings. Points in the kernel density estimates were weighted by the respective outcome feature NMF factor weights. (**C,D**) Top: the fraction of patients that were dominant for each latent factor; Bottom: stacked bar plots of the distribution of latent factor contributions across patients dominant for that factor. (**C**) Intake factors; (**D**) Outcome factors. Coloring for latent intake features: light green = degree of consciousness; light orange = severity of intracranial injury; blue = PEMHC; deep pink = PEMC; brown = sociomedical factors. Coloring for latent outcome features: light blue = functional recovery; pink = post-TBI stress; light brown = cognitive impairment; gray = 3-month neurobehavioral impairment; orange = emotional distress, yellow = concussive and episodic memory issues; lime green = vestibulo-ocular symptoms; turquoise = sleep disturbance.

We visualized the landscape across the entire patient population in terms of the latent factors at the individual patient level. Figure 3A,B visualizes individual patients projected in the two-dimensional UMAP spaces derived from the original intake (Fig. 3A) and outcome (Fig. 3B) features (see “Methods” section). Here, individual patients were plotted as pie-charts, where each pie slice is proportional to the relative weight of an intake (Fig. 3A) or outcome latent factor (Fig. 3B) to that patient's description. The localization of individual latent factors was high-lighted by computing kernel-density estimates of the factors, and displaying them around the main plot (Fig. 3Ai–v,Bi–viii). We observed that several intake characteristics and outcome phenotypes occupied distinct and localized regions of their respective landscapes, while others were more diffusely distributed across the entire plane. For the intake characteristics, for example, patients that strongly expressed intracranial injury were localized to the bottom-right (yellow, Fig. 3A, Ai) while patients that strongly expressed sociomedical factors were localized to a ridge in the upper-left (brown, Fig. 3A,Av). In contrast, the degree of consciousness characteristic (green, Fig. 3A,Aii) and pre-existing mental health conditions (blue, Fig. 3A,Aiii) were broadly distributed, indicating that they are diffusely present across patients. Notably, however, there is a subset of patients that have high intracranial damage and low consciousness (Fig. 3 A, bottom left-quadrant): these are the most severely injured patients. This visualization also suggests patterns of comorbidity expressed by individual patients. For example, a subset of patients had high expression of pre-existing medical conditions (pink) and sociomedical factors (brown) as well as a high degree of consciousness (green) (Fig. 3A middle region, Fig. 3Ai,iv,v), and another subset exhibited almost exclusive comorbidity of sociomedical factors (brown) and degree of consciousness (green) (upper right-hand ridge, Fig. 3A,Ai,iv). Compared to the intake characteristics, we observed more overlap in the expression of the outcome phenotypes at the individual patient level. That is, generally speaking, individual patients were comorbid for multiple outcome phenotypes, and the outcome phenotypes themselves occupied overlapping regions of the space (Fig. 3B,Bi–viii). For example, patients in the middle of the space expressed comorbidity of several outcome phenotypes. However, a few phenotypes were relatively localized, such as post-TBI stress in the upper region (pink, Fig. 3B,Bii), and high expression of functional recovery of patients in the lower region (blue, Fig. 3B,Bi).

We summarized the dominance and comorbidity of patient intake characteristics and outcome phenotypes across the population. We first grouped patients according to their most heavily weighted latent factors (i.e., the largest pie slice in Fig. 3A,B) resulting in five intake characteristic groups (Fig. 3C) and eight outcome phenotype groups (Fig. 3D). For each group, we then averaged the latent factor contributions to the patients to create a summary representation. In the top row of Fig. 3C,D, we plot the proportion of patients that were dominant for each factor, while the bottom row displays stacked bar plots of the distribution of factor contributions for the patients dominant in that factor. Reinforcing the results described above, we found that, for the intake characteristics (Fig. 3C), the majority of patients (81.0%) were dominant for the high degree of consciousness. On average, in these patients, the other four characteristics were present in roughly equal proportions. A smaller, yet notable portion (15.0%), was dominant in the intracranial injury factor, which was modulated mostly by degree of consciousness characteristics. Some patients were dominant in the PEMC and PEMHC, while no patients were dominant in sociomedical factors (depicted with a hatched bar). Likewise, for the outcome features (Fig. 3D), the majority of patients are high in functional recovery (84.6%), consistent with the observation that the majority of patients had mild TBI characteristics (Fig. 3C). A smaller portion (14.6%) was dominant in the post-TBI stress factor, while a much smaller fraction of patients were dominant in the expression of cognitive impairment, 3-month neurobehavioral impairment (NBI), as well as emotional distress phenotypes. No patients were dominant in vestibulo-ocular, sleep disturbance and episodic memory factors. Broadly speaking, the patterns of comorbidity in these groups were consistent with the patterns observed at the individual patient level. For example, the patients that most strongly expressed PEMHC (blue) also expressed comorbidity with high degrees of consciousness (green) characteristics (Fig. 3C). Likewise, with the notable exception of the functional recovery phenotype, the patients that were dominant in the expression of an outcome phenotype were comorbid for many other outcome phenotypes (Fig. 3D). There was no significant Spearman correlation between the number of patients dominant in a factor and the number of features in that factor (*P* > 0.1). Together, these results demonstrate the quantification of qualitatively known patterns of dominance and comorbidity, as well as revelation of new patterns. They thus highlight the utility of the holistic latent factors in quantitative description of single patient intake characteristics and outcome phenotypes.

### TBI patient outcomes can be predicted with a high degree of precision

The results above demonstrate that individual TBI patients are described by multiple intake characteristics and can end up with diverse combinations of outcome phenotypes. Although individual TBI patients are typically classified into a small number of outcome phenotype categories (e.g., mild, moderate, severe), the analysis presented above suggests such course descriptions likely mask underlying complexity of the patient outcome symptomatology. Here, we take a data-driven approach to determine how many ‘types’ of TBI outcomes are present in the TRACK-TBI Pilot data.

We developed a novel approach to determine how many types of TBI outcomes can be predicted from the intake data through a combination of non-parametric unsupervised and supervised machine learning approaches (see “Methods” section). The intuition behind our method is that classification accuracy (relative to chance) should plateau at the true number of predictable clusters in the dataset. Briefly, we first perform unsupervised hierarchical clustering on the outcomes, and systematically vary the number of detected clusters. We then train a Random Forest classifier to predict patient outcome cluster membership from intake features for various number of outcome clusters. Note that the classification problem gets harder as the number of classes (i.e., outcome clusters) increases—that is, classification accuracy will generally decline as the number of classes increases. Thus, we normalized the predictive accuracy of the classifier by the data-derived chance accuracy, to arrive at the Fold Over Chance (FOC) metric. We determine the number of clusters in the data as the estimated asymptote of FOC as a function of the number of clusters. In synthetic data, we found that this method was highly accurate, with a linear relationship between true and estimated number of clusters accounting for 98% of the variance in the estimated number of clusters (R^2^ = 0.98, Supplementary Fig. 5). Thus, we have developed a simple, non-parametric, data-driven approach to determine the number of outcome clusters that can be predicted from the intake features.

**Figure 4:**
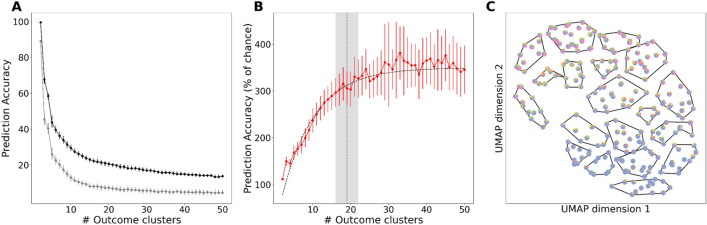
TBI patient outcomes can be predicted with a high degree of precision. (**A**) Accuracy of predicting outcome clusters using clinical features for different numbers of clusters. Each point corresponds to clustering outcomes into a different number of clusters. Error bars on each point represent the standard deviation of accuracy across UMAP replicates. Black line corresponds to true predictive accuracy and gray line corresponds to chance predictive accuracy. (**B**) Fold-over-chance accuracy of predicting outcome clusters using clinical features for different numbers of clusters. Each point corresponds to clustering outcomes into a different number of clusters. Error bars on each point represent the standard deviation of accuracy across UMAP replicates. Fold-over-chance values were calculated as the ratio of true predictive accuracy to chance predictive accuracy. Black dotted line represents the least squares exponential fit. Vertical gray dotted line represents estimate of number of clusters and gray shading represents 95% CI around estimate. (**C**) Two-dimensional UMAP embedding clustered into 19 clusters. Points are drawn as pie charts calculated using normalized outcome NMF weights.

We utilized the method described above to estimate the number of TBI outcome clusters that can be predicted from the intake features. Figure 4A plots the raw classification accuracy for predicting outcome cluster membership from intake features as a function of increasing number of outcome clusters (black, mean ± s.d., N = 250 non-parametric bootstrap samples), as well as the data-derived chance accuracy (gray, mean ± s.d., N = 250 non-parametric bootstrap samples). As expected, both raw classification accuracy (black) and chance performance (gray) declined monotonically as the number of clusters increased. In contrast, Fold Over Chance (FOC) increased to an asymptote (Fig. 4B, red points, mean ± s.d., N = 250 non-parametric bootstrap samples). The dashed black line in Fig. 4B is the best fitting asymptotic function. Using the procedure described above, we estimated that the number of TBI outcome clusters was 19 ± 2 (Fig. 4B, mean ± 95 C.I. dashed vertical line and gray region). For 19 outcome clusters, the median raw classification accuracy was 0.210, while the median chance accuracy was 0.0726 (median FOC was 3.06, i.e., 306% of chance). We visualized how the 19 clusters grouped individual patients relative to their outcome latent factor compositions. Figure 4C plots the 247 patients (represented as outcome latent factor pie charts, as in Fig. 3B) in the 2D UMAP outcome space, with bounding polygons around the patients in the same cluster. The 19 clusters tiled the outcome landscape, and the latent factor composition (pie charts) associated with the patients within the same cluster were relatively homogenous compared to the entire population. For example, the gray-shaded cluster at the bottom was composed of patients dominated by the functional recovery latent factor, while patients in the gray-shaded cluster were dominated by post-TBI stress latent factor (albeit with contributions from other factors, as expected). These results indicate that the predictive clustering of outcomes grouped together patients with similar outcome phenotypes (see also Supplementary Fig. 6). Together, these results demonstrate that there is an unappreciated degree of outcome phenotypic precision that can be predicted from the intake features in deeply phenotyped TBI patients.

### Holistic individual patient outcomes can be predicted from intake features

**Figure 5:**
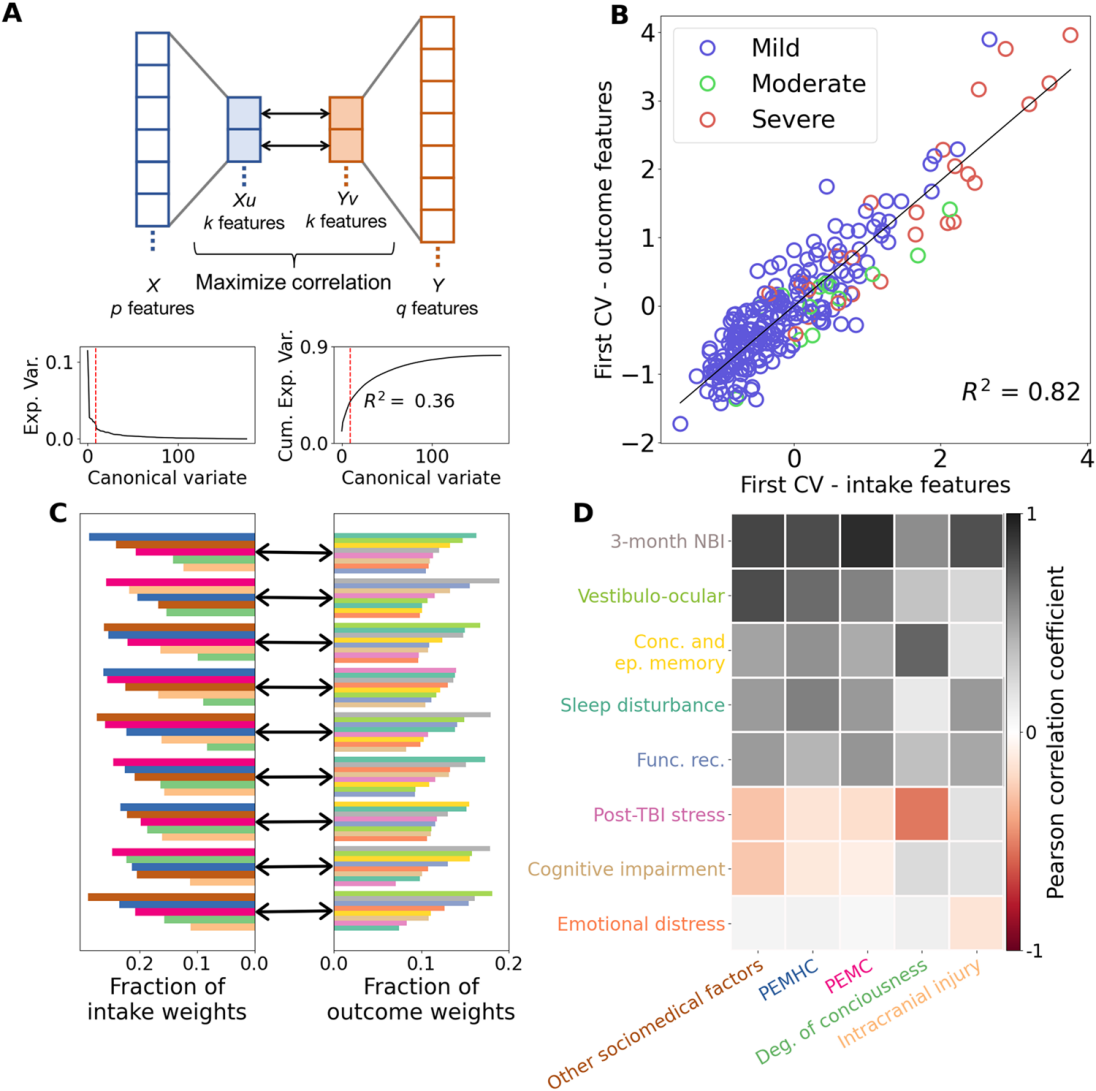
Holistic individual patient outcomes can be predicted from intake features. Sparse canonical correlation analysis of clinical feature and outcome data. (**A**) Upper. Canonical correlation analysis projects observed features into maximally correlated latent spaces. Lower. Outcome variance explained by canonical variates (left) and cumulative outcome variance explained by canonical variates (right). Dotted red line indicates cutoff for significant canonical variates. (**B**) First canonical variate. Solid black line indicates least squares regression line with corresponding *R*^*2*^ indicated on plot. Points are colored according to the patient Glasgow Coma Scale classification. (**C**) Absolute total leverage of NMF features within each canonical variate. (**D**) Predictive relationship between intake characteristics and outcome phenotypes. Color bar depicts the Pearson correlation coefficient.

The results presented above indicate there are many more types of TBI outcomes that are predictable from intake data than previously known. We next sought to determine the degree to which holistic patient outcomes can be predicted from intake features at the individual patient level. Here, our goal is to provide a holistic prognostic model by predicting as much of the full heterogeneity and complexity of individual patient outcomes as possible from the intake data, while retaining interpretability. To this end, we deployed sparse canonical correlation analysis (sCCA), a method for simultaneously computing aggregations of features for two separate sets of data that are maximally linearly correlated with each other. Briefly, sCCA computes paired sets of canonical variates (CVs)—in this case one set of variates each for the intake and outcome features. Each pair of CVs is a (sparse) weighted combination of features in one set (i.e., intake features) that are maximally correlated with a (sparse) weighted combination of features in the other set (i.e., outcome features) (Fig. 5A, upper). We derived equations that enabled us to calculate how much of the total outcome variance could be explained by each CV (see “Methods” section), and ordered them according to explained outcome variance.

When applied to the TRACK-TBI Pilot data, we found that the amount of explained outcome variance decreased quickly as a function of the number of CVs, and exhibited a long tail after nine variates (Fig. 5A, lower left). This indicates that the CVs beyond nine provide diminishing returns on explained outcome variance. These first nine variates (5% of the total 177 CVs) explained 36% of the total variance across individual patient outcomes (Fig. 5A, lower right) (R^2^ = 0.36 > 17 s.d. from mean of null distribution; *P* < 10^−3^, N = 247 patients, Permutation test, Supplementary Fig. 7). As an example of the predictive relationships captured by the canonical variates (CVs), in Fig. 5B we plotted each patient in the plane formed by the first CV (x-axis: 1st intake canonical variate; y-axis: 1st outcomes canonical variate, black line is best linear fit). Here, each patient is colored according to the severity of their TBI as assessed at intake (mild, moderate, severe, Fig. 5B). We found that the cross-validated predictive accuracy was very high, with 82% of the variance in the first outcome CV accounted for by the first intake CV (*P* < 10^−10^ N = 247 patients). Note that this predictive accuracy is restricted to just the outcome variance associated with the first canonical variate, which itself accounted for a fraction of the total outcome variance (see Fig. 5A, bottom). Additionally, there was a visually apparent ordering of TBI severity contained in the predictive relationship captured by the CVs: mild injuries were distributed predominantly at the lower end of the numerical range, moderate injury in the middle range, and severe injuries at the upper range. Similar, though more complex, relationships were observed when each patient was represented with the intake and outcome latent factor contribution pie-charts (Supplementary Fig. 8). Together, these results demonstrate that a small number of canonical variates (9/177, 5%) can explain a sizable fraction (36%) of the total outcome variability across individual TBI patients that are deeply phenotyped, and CVs retain clinically meaningful structure towards prediction of TBI outcomes from intake variables.

The canonical variates (CVs) were highly distributed across the patient population, in contrast to the NMF latent factors (see Supplementary Fig. 9). Thus, to gain deeper, more holistic insight into the contributions of intake characteristics towards prediction of outcome phenotypes, we analyzed the CVs through the lens of the NMF latent factors (see “Methods” section). Briefly, the latent factors describe weighted combinations of intake/outcome features that parsimoniously account for the structure of the full data. Similarly, the canonical variates identify weighted combinations of intake features that are maximally predictive of weighted combinations of outcome features. Thus, we can combine those weights to examine how combinations of intake characteristics are predictive of combinations of outcome phenotypes. In Fig. 5C, we plot the fractional contributions of the latent intake characteristics and outcome phenotypes for each of the nine canonical variates. We observed that both the intake and outcome CVs were composed of non-uniform contributions of latent factors. For example, the first canonical variate emphasized how the combination of pre-existing mental health conditions (PEMHC, blue) and sociomedical factor (brown) intake characteristics were predictive of sleep disturbance and vestibulo-ocular outcome phenotypes (Fig. 5C, top row). Across CVs, we observed that different patterns of intake characteristics were predictive of different patterns of outcome phenotypes. We summarized the predictive relationships between the latent factors by computing the cross-covariance for the NMF factors projected into the canonical variates (Fig. 5D). We found that the strongest relationships were between presentation of pre-existing medical conditions (PEMC) contributing to 3-month neuro-behavioral impairment (NBI), and higher degree of consciousness contributing to less post-TBI stress. (Fig. 5D, colorbar). We also observed remarkable consistency in the relative importance of pre-injury intake phenotypes (sociomedical factors; PEMHC; PEMC) for predicting multiple outcome phenotypes with moderate correlation strength (3-month NBI; vestibulo-ocular; concentration and episodic memory; sleep disturbance; functional recovery). In contrast, the intake phenotypes comprising objective indicators of clinical injury emerged as strong predictors of specific outcome phenotypes (degree of consciousness for post-TBI stress, and concussion and episodic memory; intracranial injury for 3-month NBI). Likewise, we found a gradation in the extent to which outcome phenotypes could be predicted, with 3-month NBI being the most predictable, while emotional distress and cognitive impairment being the least predictable. Together, these results quantify the complexity by which combinations of intake characteristics lead to outcome phenotypes.

## Discussion

Traumatic brain injury is defined as an injury to the brain resulting from an external force^21^. This injury interacts with individual patient backgrounds to result in complex and heterogeneous outcome phenotypes. Understanding and predicting holistic outcome phenotypes with high precision from intake data is a major challenge in the field. To address this gap, we developed novel combinations of unsupervised and supervised machine learning algorithms and applied them to a cohort of deeply phenotyped patients in the TRACK-TBI Pilot data set. We found that the complexity of the data could be distilled to a small number of latent factors associated with clinically meaningful intake characteristics and outcome phenotypes. Furthermore, we found that ~ 19 types of TBI outcome clusters can be predicted from the intake features, an ~ 6× improvement in precision over the current clinical standard of care (mild, moderate, and severe TBI). Finally, 36% of the total variance across individual patients could be predicted from a small number of latent predictive mappings between the entire intake and outcome features.

### Data-driven distillation of complex intake characteristic and outcome phenotype data

Every person's medical trajectory is defined by the entirety of their life experience, including their pre-existing conditions and socioeconomic status. In the brain, interacting but distinct anatomical regions are responsible for performing different functions. For example, the hippocampus is important for formation of episodic memories^22,23^. As such, in TBI there are a diverse set of outcome phenotypes that can occur, depending on the interaction of the patient's pre-injury status with the location and severity of the brain injury^24–26^. Precision medicine requires not only capturing this diversity through deeply characterizing individual patients, but understanding and leveraging this knowledge towards holistic, personalized predictions and treatments. However, such precision medicine data, which represents the clinical knowledge about the patients, is often very complex: individual patients are associated with a large number of both intake and outcome features. This complexity often presents an impediment to clinical understanding. In the context of the patients composing the TRACK-TBI Pilot study, we focused on the subset of patients that had the most complete set of a large number of intake and outcome features. This was done to maximize the amount of information present in both the intake and outcome feature spaces.

We sought to provide data-driven, holistic insight into the entirety of intake and outcome features that describe individual patients through interpretable unsupervised machine learning methods. We found that our UoI-NMF algorithm distilled the complexity of the high-dimensional original features down to a small-number of clinically meaningful concepts associated with intake characteristics and outcome phenotypes. The discovered latent factors grouped related original features together, thus naturally handling issues of multicollinearity in the data. Therefore, we have defined latent intake characteristics and outcome phenotypes, and the individual items they are composed of, to effectively provide data-driven distillation of complex TBI knowledge that has eluded researchers for the past 4 decades.

The latent factors are composed of many original features that co-occur across patients, and each patient can be described as weighted combinations of those latent factors. Importantly, this enables quantitative description of individual patients' expression of the holistic intake characteristics and outcome phenotypes (e.g., Fig. 3). Within the patient population that is alive/non-comatose at 6 months, there are many dimensions that differentiate their quality of life, and patients exhibit a spectrum ranging from fully recovered to severely debilitated^8^. We found that individual patients can be quantitatively mapped by their intake and outcome phenotypes to show the relative contribution of each latent phenotype to each patient’s presentation, as well as the co-occurrence of distinct intake and outcome phenotypes in clusters of patients. For example, degree of consciousness and functional recovery are diffusely enriched across the majority of patients (reflecting that most patients had mild TBI and recovered), post-TBI stress co-occurs with a subset of PEMHC and sociomedical factors, and severe intracranial injury and sociomedical factors tended not to co-occur. These data-driven revelations make intuitive sense, as the predominance of TBI patients in TRACK-TBI Pilot were on the milder end of the severity spectrum from concussion to coma, which is dependent on assessment of functional recovery, and of initial Glasgow Coma Scale (GCS) score. Further, post-TBI stress is known from literature to be associated with pre-existing mental health conditions and sociomedical factors^27^; however the precise relationships are complex. The emergence of intake phenotypes with little overlap (sociomedical factors; intracranial injury) highlight that the component features of these two latent phenotypes weigh differently across subpopulations of patients, which can enable identification of these subpopulations during classification approaches. Together, these results demonstrate the power of interpretable unsupervised machine learning for data-driven distillation of complex precision medicine data into clinically meaningful, quantitative descriptions of individual patient intake characteristics and outcome phenotypes.

### From categories to continuums towards precision prognostication

The promise of personalized treatments from precision medicine is predicated upon the ability to accurately and precisely predict patient outcomes from intake data. Furthermore, it is desirable for the outcome predictions to be in the context of holistic descriptions of patients that account for multiple phenotypic dimensions, not just single outcome measures. Put another way, the ability to tailor treatments for individuals requires the ability to predict patient outcomes with high accuracy and high precision across the diversity of phenotypic domains. There is typically an inherent tradeoff between precision and accuracy, with more precise parcelations of an outcome (e.g., increased number of outcome classes/clusters) making accurate prediction more difficult. For example, predicting mortality and/or severe disability at 6 months following TBI requires comparatively less precision than predicting performance on complex measures of functional, cognitive, and neuropsychiatric recovery, as evidenced by well-validated prognostic calculators based on large multicenter datasets^28,29^. Conversely, we argue that increased outcome precision is naturally associated with more holistic outcome descriptions, as the ability to meaningfully describe individuals with increased precision requires more diverse types of information about those individuals to be taken into account. We distilled this diverse information into latent factors. Indeed, the latent factors associated with outcome phenotypes enables quantification of an individuals' expression of combinations of those outcome phenotypes, providing holistic and precise descriptions. However, these outcome phenotype descriptions do not speak to the ability to predict whether a patient will exhibit those outcomes. That is, how precisely can we prognose patient outcomes?

In the subset of deeply phenotyped patients we focused on in the TRACK-TBI Pilot data, we found that there were ~ 19 clusters of TBI outcomes that can be predicted from the intake data. Individual classes were composed of patients with similar combinations of multiple outcome phenotypes^30^. For 19 outcome clusters, the median raw classification accuracy was 21%, which was 306% of chance. While this accuracy is not likely to reach the level of clinical actionability, we note that the subset of patients associated with the large number of features necessitated by our goals strongly suggests that increasing the patient population with the same degree of deep phenotyping will increase prediction accuracy, and perhaps outcome phenotype precision as well. Nonetheless, these results demonstrate a high-degree of previously unknown precision of predictability of patient outcomes.

The results described above indicate many more post-injury clusters of TBI are predictable from intake data than previously appreciated. This in-and-of-itself is a major step toward precision description and prediction of TBI outcomes. However, it still treats the patients in a cluster as undifferentiated members of a homogenous group, not as individuals. Conceptually, carrying prediction precision to its logical conclusion by increasing the number of clusters until we attempt to predict the precise outcomes of each individual patient from intake data turns the classification problem to a regression problem. Typically, individual patient predictive modeling is done by considering single outcome variables^31^. In contrast, our goal was to predict as much of the full heterogeneity and complexity of individual patient outcomes as possible to provide a holistic and complete prognostic model. We found that 36% of the *total* variance across all outcome features at the individual patient level could be predicted from intake features with nine canonical variates. To the best of our knowledge, no prior study has attempted to explain the total variance across all outcome variables. As such, the sheer novelty of this finding makes it significant. To view the predictive mapping through the lens of the distilled intake characteristics and outcome phenotypes, we leveraged the compositionality of linear methods (i.e., NMF and CCA). This revealed novel insights into how the complex interaction of pre-existing conditions and injury severity give rise to diverse outcome phenotypes.

Here, we utilized predictive accuracy and precision as lenses with which to quantitatively interrogate the relationships between intake characteristics and outcome phenotypes, but it is not the end goal– the end goal is precision prognosis. In the specific context of TBI, prognostication has been hampered by lack of accuracy and precision, without validated prediction models beyond acute mortality, 6-month mortality and unfavorable outcome^28^. Extant prediction models rely on multivariate regressions of presentation and injury variables onto dichotomized, multiclass, or ordinal prediction of single outcome variables , which inherently limits precision^13,32^. Furthermore, prognosis calculators acceptable to the clinical community have been limited to global outcomes, such as the Glasgow Outcome Scale—a 1–5 or 1–8 ordinal scale of outcome from death to full recovery. The lack of acceptable prediction models for more granular outcomes and outcome domains has been limited by the heterogeneity of: (1) pre-existing medical and social condition and injury severity, (2) treatment course and interventions, and (3) symptomatology constituting multidimensional outcomes. Critical to the absence of a precise prediction model is the lack of accurate and precise definitions of “clinically important” injury intake and outcome factors suitable for measurement across the spectrum of TBI, which has limited the advancement of TBI care. Our solution to this pressing issue is to view predictive models through the lens of the latent intake characteristics and outcome phenotypes. The importance and relevance of our approach is that it was driven by the inherent heterogeneity, collinearity, and noise of the presentation and outcome data in each unique TBI patient presenting to care (i.e., data-driven). For the first time, distillation and capture of these latent intake and outcome phenotypes more certainly *defines* the relevant underlying phenotypic categories, which have escaped capture in historical approaches, and can be targeted for precision prognosis. Equally important is our distillation of the various individual outcome symptomatology scores to reduce the inherent collinearity and redundancy between similar symptoms assessed by different measurement tools—data parsimony. This is requisite to enabling precise prognosis. See Supplementary Discussion for more in-depth discussion on TBI specific metrics. If this approach can be validated and adopted in the clinical realm it will obviate the historical limitations to defining a “clinical state” and truly advance our ability to perform precision prognostication.

### Data collection and methodological considerations for data-driven precision prognostication

The ability to detect the high precision found here depended on the combination of the development of statistical-machine learning algorithms and the application of those methods to deeply phenotyped patients. Put more precisely, the entropy (i.e., heterogeneity) of features in both the intake and outcome data put mathematical limitations on the precision with which predictions can be made. A corollary of this is that fewer outcome types would (likely) be detectable in datasets (either other datasets or larger subsets of the TRACK-TBI Pilot data) that have fewer features associated with each patient.

Inevitably, as more features are collected on patients, there will be overlap in the information captured by those features. A recurring challenge for many traditional approaches to the analysis of complex biomedical datasets is the presence of multicollinearity (i.e., correlations) amongst the features. For example, in the TRACK-TBI Pilot data set, several features are rescalings of other features, or are composites of several features. The latent factors discovered here naturally deal with this by grouping collinear features together. Indeed, having collinearity amongst features is required to reduce their dimensionality. Likewise, the methods we used for predictive modeling are less sensitive to collinearity (Random Forests), or directly take it into account (CCA). Generally speaking, modern statistical-machine learning algorithms have become more robust to the presence of multicollinearity in datasets. But, if a clinical feature is not collected, that information is forever lost. Thus, we advocate for the collection of ever more detailed and deeply phenotyped biomedical datasets. At the same time, we appreciate that there is a cost associated with all data collection, and given the finite resources available, this necessitates a tradeoff between the size of the patient cohort examined and the depth of phenotyping that can be achieved. One approach would be to conduct modest size studies with as much phenotypic depth as possible, and then use data-driven approaches (e.g., the NMF feature weightings) to identify the most important variables to collect in future large-scale studies.

Our analysis depends on having a large number of intake and outcome features for each patient, and this dependence resulted in a sub-selection of N = 247 patients. Removal of missing data can introduce bias in medical study results since there are often nontrivial reasons for data missingness in clinical data collection. One could use stochastic missing value imputation to address this issue. UMAP can reproduce different (sometimes discordant) results with multiple resampling runs. However, we did not observe this in our analysis. We sought embeddings that preserved both global and local structure, though empirical hyperparameter selection could be performed. Furthermore, there was substantial racial disparity in the data set, with mostly white patients, which may result in challenges generalizing to other populations. Finally, we focused on those patients with a GOSE > 2, as those patients had the most robust features, and were the most poorly understood. As such, while our results are highly statistically significant and use cross-validated predictive accuracy as a metric, future studies should increase the sample size to ensure predictive generalizability.

One of our goals was to provide data-driven insight into the complex TRACK-TBI Pilot data using methods that are amenable to the modest sample size and are easily deployable and scalable. For example, we utilized the compositionality of unsupervised (NMF) and predictive (CCA) linear models to provide quantitative insights into predictive models. We used a novel combination of standard non-parametric clustering and predictive models for discovery of the number of outcome clusters. An alternative would have been to utilize Bayesian methods; however, those approaches typically require more complex models, and scalability of inference can be a challenge. Likewise, while deep networks could be used to maximize both prediction accuracy and precision, the highly non-linear nature of deep networks make them hard to interpret, and the large number of parameters make them prone to overfitting. Exploring how to utilize such approaches towards interpretable distillation of complex data and improved prognostic precision given the data constraints is an area of active research. Finally, conformal analysis should be deployed for confidence in clinical decision making.

In many clinical settings, an individual's medical outcome is the result of interactions between the environment that they are exposed to and the cause of the condition. For example, even in Huntington's Disease, which has a known genetic cause, there exists extreme heterogeneity in the onset of symptoms for low-penetrance genotypes^33^. This heterogeneity is thought to arise from the diversity of environments that those patients live in, but is poorly understood. In the context of TBI, we have demonstrated distillation and quantification of how a patient's complex outcome phenotype depends on the combination of their injury characteristics with pre-existing conditions, some of which may have social determinants. Therefore, we contend that the approach taken here for TBI will be applicable to complex biomedical datasets associated with many other health conditions.

## Methods

### Study and subjects

In the early 2000s, the National Institutes of Health (NIH) and National Institute of Neurological Disorders and Stroke (NINDS) developed the TBI Common Data Elements (CDEs) to overcome long-standing pitfalls in TBI clinical research, including lack of standardization in data collection and analysis, inability to appropriately stratify patients, and disparate injury types^34^. Using a consensus-based approach, in 2010 NINDS working groups established standards for data capture across 4 broad domains: clinical assessments and demographic information, genetics and proteomics, neuroimaging, and outcome measures^35–38^. The prospective, multicenter, observational TRACK-TBI Pilot assessed the feasibility and utility of version 1 of the TBI CDEs^7^, setting the stage for feedback to the NINDS working groups, refinement of the CDEs^39^, and subsequent large-scale multicenter prospective efforts in the US and Europe^40^.

TRACK-TBI Pilot was conducted at three U.S. Level I trauma centers (Zuckerberg San Francisco General Hospital (California, US), University of Pittsburgh Medical Center (Pennsylvania, US), University Medical Center Brackenridge (Texas, US)). Inclusion criteria were patients of all ages with external force head trauma and presentation to a participating institution with a clinically indicated head computed tomography (CT) scan within 24 h of injury. Exclusion criteria were pregnancy, ongoing life-threatening disease (e.g., end-stage malignancy), police custody, involuntary psychiatric hold, and non-English speakers due to multiple outcome measures administered and/or normed only in English. Institutional Review Board (IRB) approval was obtained at each participating institution with the University of California, San Francisco (UCSF) as the coordinating center (UCSF Committee on Human Research, Study #10-00011). Informed consent was obtained from each subject prior to enrollment. For patients unable to consent for themselves, the informed consent process was pursued with the surrogate next of kin or legally authorized representative. Consent for patients under 18 years of age was obtained from the parent or legal custodian and accompanied by patient assent if the patient was 7 years of age or older. Subjects were re-consented, if cognitively able, during the course of clinical care and/or follow-up timepoints for study participation. See Yue et al.^7^, for further details.

Between April 2010 and May 2011, TRACK-TBI Pilot enrolled 599 acute TBI patients, and final outcomes testing was completed in June 2012; 13 subjects aged < 16 years were excluded due to differences in variables recommended by CDE working groups, resulting in 586 subjects suitable for legacy analyses. At the time of TRACK-TBI Pilot implementation, 6 months post-injury was regarded as the gold standard time-point for outcomes assessment by CDE working groups and a comprehensive battery of 10 representative measures covering the consensus-selected domains of global outcome, neuropsychological impairment, psychological status, post-concussive symptoms, social role participation, and quality of life were administered (Glasgow Outcome Scale-Extended [GOSE]; Neurological Symptoms Inventory (NSI); California Verbal Learning Test, Second Edition; Wechsler Adult Intelligence Scale, 4th Edition-Processing Speed Index; Trail Making Test; PTSD Checklist-Civilian Version; Brief Symptom Inventory-18 Item; Rivermead Post Concussion Symptoms Questionnaire; Craig Handicap Assessment and Reporting Technique-Short Form; Satisfaction with Life Scale)^37^; at 3 months, a subset of measures (GOSE, NSI) were administered as part of evaluating the feasibility and utility of the 3-month time-point, as previously described^7^. In the current study, patients and features were excluded to remove missing values in the intake and outcome feature matrices. The resulting dataset had 247 patients with 235 intake features and 177 outcome features. Of the 586 total patients, the number of patients with outcome data from the original dataset are: 3 months, 463; 6 months, 418; 12 months, 280. The number of patients with outcome data from filtered dataset: 3 months, 247; 6 months, 247; 12 months, 0.

The GCS was obtained upon arrival to the emergency department by the treating clinician in the emergency department. Demographic, socioeconomic, and medical history variables were collected directly from the patient and/or caregiver when available, as well as from chart review of the medical record. All treatment data up until hospital discharge were extracted directly from the medical record. Outcome data were obtained from patient interviews, which were conducted in person at 6-months post-injury and by telephone at 3-months post-injury. See Yue et al.^7^, for further details.

### Glasgow coma scale classification

Glasgow coma scale (GCS) was divided into subranges for visualization and cross-validation stratification. The subranges for the three classifications are: GCS 3–8, severe; GCS 9–12, moderate; GCS 13–15 mild.

### Subject selection criterion and feature cleaning

The TRACK-TBI Pilot dataset used for analysis was unusable in its raw form. All patients had at least one missing intake variable and one missing outcome variable, and 76% of all intake variables had at least one patient missing while all outcome variables had at least one patient missing. In aggregate, 25% of the original intake data had missing values, while 51% of outcome data had missing values. To avoid imputing data, these missing data points were addressed by removing patients and intake and outcome variables to produce a dataset with no missing data. This resulted in a final population size of N = 247. Finally, categorical features were one-hot encoded: i.e., create a dummy variable for each category. The software and raw data are available in the Github repository for this paper (see below). The racial breakdown of the 247 patients is as follows: 192 Caucasian, 23 African American or African, 11 Asian, 8 Hawaiian or Pacific Islander, 8 More than One Race, 3 American Indian or Alaskan. There were 174 males and 73 females.

### Software and data

All software and data are freely available at: https://github.com/BouchardLab/ML_4_prec_prognosis and https://github.com/TRACK-TBI-Public.

### Statistical tests and hypothesis testing

We deployed resampling techniques (i.e., cross-validation, bootstrap resampling, see below for details) for the metrics of interest so as to produce confidence intervals and perform statistical tests. Statistical tests were deemed as significant if the probability of incorrectly rejecting the null hypothesis was alpha < 0.05.

### Non-negative matrix factorization via union of intersections

Non-negative matrix factorization (NMF)^41^ was carried out using the Union-of-Intersections-NMF (UoI-NMF)^42^ framework*. THE UoI-NMF algorithm utilizes bootstrap sampling when deriving the latent representations*^42^*. This approach greatly reduces sensitivity to perturbations in patient sampling and data values.* UoI-NMF was executed using the *pyuoi* (version 1.0.0) Python package^43^. Before executing UoI-NMF, NMF hyperparameters (number of latent features and elastic-net^44^ regularization parameters) were optimized. The multiplicative-update algorithm was used to solve the Kullback–Leibler divergence loss function^45^. For hyperparameters, tThe number of latent factors was chosen as the number of factors that minimized the Bayesian Information Criterion (BIC)41. Regularization parameters (regularization strength and L1-ratio) were chosen by visualizing the bases from factorizations calculated for all pairwise combinations of the following parameter sets: regularization strength of [0.01, 0.1, 0.2, 0.5, 1.0, 2.0, 5.0] and [0.1, 0.2, 0.5, 1.0, 2.0, 5.0] for outcomes and biomarkers, respectively, and L1-ratio of [0.1, 0.2, 0.5, 0.7, 1.0] for both outcomes and biomarkers. Each factorization was visually inspected, and the factorization that appeared to produce the most disjoint factors, in terms of loadings from the original features, was chosen. After NMF hyperparameters were determined, UoI-NMF was run. To ensure UoI-NMF produced the desired number of latent factors, the DBSCAN ^46^ clustering step of UoI-NMF was replaced with K-means^47^. K-Means and NMF were run using Sci-Kit Learn (version 0.23.1)^48^.

### UMAP

Embeddings calculated with the Uniform Manifold Approximation and Projection (UMAP) method^49^ were executed using the *umap-learn* (version 0.3.10) Python package (https://github.com/lmcinnes/umap). Clinical feature embeddings were calculated using default parameters with the exception of the following parameters: *n_components* = 2, *min_dist* = 1.0, *random_state* = 20,001. Outcome embeddings were calculated using default parameters with the exception of the following parameters: *n_components* = 2, *min_dist* = 0.0, *random_state* = 20,001. Additionally, we used a custom distance matrix to accommodate the high proportion of binary features in the outcome features. This distance matrix was calculated as a weighted average of distance matrices computed for binary and continuous features. The distance matrices for binary and continuous features were computed using the Jaccard–Needham dissimilarity and the Euclidean distance, respectively.

### Number of outcome categories

The number of outcome categories was determined by calculating the smallest number of outcome clusters that achieved the maximum predictive accuracy when regressed onto the clinical features. After computing an upper limit on predictive accuracy, the outcome clustering with the fewest clusters that achieves the maximum predictive accuracy within some level of uncertainty. Before clustering, outcome variables were embedded into a low-dimensional space using UMAP^49^ (*n_components* = *2, min_dist* = 0.0*, random_state* = 20,001). The min_dist parameter was set to 0.0 to create more compact clumps to improve downstream clustering analysis, and n_components was set to 2 to reduce computational complexity after we verified that this parameter did not affect downstream results. After embedding with UMAP, patients were then clustered with Ward’s method^50^ for agglomerative hierarchical clustering using low-dimensional embeddings produced by UMAP. The threshold was varied to produce clusterings with 2 to 50 clusters. These clusterings were evaluated by calculating the cross-validated predictive accuracy from regressing cluster labels onto the patient clinical feature data using random forests^51^. For cross-validation, we used five splits stratified by cluster label with no repeats. These accuracy scores were adjusted to account for the varying classification difficulty across clusterings by dividing by the cross-validated predictive accuracy of a naive classifier that was trained by regressing randomly shuffled cluster labels onto patient clinical feature data using random forests and the same cross-validation procedure used in the previous step. This gives us the fold-over chance predictive accuracy (FOC). This entire process was carried out 250 times to account for instability in clustering results from stochasticity in UMAP embeddings and the mean FOC and standard deviation was calculated for each number of clusters. To determine the upper limit on FOC i.e. maximum predictive accuracy, we modeled predictive accuracy as a function of the number of clusters. Using this function, we take the limit as the number of clusters approaches infinity to find the maximum predictive accuracy. To do this, we fit an exponential function to the FOC scores across all clusterings. The function takes the form $A = x_{0} + x_{1} e^{{x_{2} C}}$, where $A$ is the adjusted cross-validated predictive accuracy, $C$ is the number of clusters in each clustering, and $x_{0}$ is the FOC upper limit. To account for uncertainty in the estimate of the upper limit of FOC and FOC for each cluster, we adjust mean calculations by their respective standard deviations. The final number of clusters was determined to be the smallest number of clusters for which the $\hat{\mu }_{A} + \hat{\sigma }_{A} > \hat{x}_{0} - \hat{\sigma }_{{x_{0} }}$, where $\hat{\mu }_{A}$ and $\hat{\sigma }_{A}$ are the estimated mean and standard deviation, respectively, of FOC scores for a clustering $A$, $\hat{x}_{0}$ is estimate for $x_{0}$, and $\hat{\sigma }_{{x_{0} }}$ is the standard deviation for the $x_{0}$ estimate.

### Sparse canonical correlations analysis

The *truly alternating least squares* algorithm presented in^52^ for solving sparse canonical correlation analysis was implemented in Python. Inner lasso^53^ regression problems were solved using coordinate-descent as implemented in Sci-Kit Learn^48^. Cross-validated explained outcome variance was used to select optimal regularization parameters. Cross-validation used five folds, stratified by patient GCS classification (i.e., severe, moderate, mild). Total outcome variance explained by a canonical variate $i$, $\eta_{i}$, is calculated by scaling the weighted sum of the cosine similarities between the outcome canonical variate and all principal components of the outcomes by the ratio of shared variance between the clinical feature and outcome canonical variate. That is:

$$\eta_{i} = r\left( {u_{i} ,v_{j} } \right)^{2} \times \mathop \sum \limits_{j} \lambda_{j} S_{c} \left( {v_{i} ,w_{j} } \right)$$

where $r$ is Pearson’s correlation coefficient, $u_{i}$ is the left (i.e., intake feature) canonical variate, $v_{i}$ is the right (i.e., outcome) canonical variate, $\lambda_{j}$ is the explained variance for outcome principal component $j$, $w_{j}$ is outcome principal component $j$, and $S_{c}$ is the cosine similarity.

### Canonical variate visualizations

Bar-plots were generated by collapsing canonical variate loadings according to nonnegative factors. The nonnegative factor to which each observed feature was assigned was calculated by identifying the factor for which the observed feature had the highest factor loading. The absolute value of canonical variate loadings of observed features were summed for each nonnegative factor. This process created $B$, a $k \times l$ matrix, where $k$ is the number of nonnegative factors and $l$ is the number of canonical variates. This process was done for both intake variables and outcome variables, creating matrices $B_{I}$ and $B_{O}$, respectively.

Let $F_{ij} = 1$ if $argmax_{k} H_{ \cdot j} = i$, $0$ otherwise, where $H$ is the nonnegative bases matrix and $k$ is the number of nonnegative factors. Let $G$ by the row L1*-*normalized form of $F$. Then $B_{I} = G_{I} \beta$ and $B_{O} = G_{O} \theta$, where $\beta$ and $\theta$ are the canonical variate loadings for the intake variables and outcome variables, respectively. The left panel and right panel of Fig. 5C were generated from $B_{I}$ and $B_{O}$, respectively. The heatmap in Fig. 5D was generated from the matrix $P$ where $P_{ij}$ is the Pearson correlation coefficient between the row $i$ and column $j$ of $B_{I}$ and $B_{O}$, respectively.

### Supplementary Information

Supplementary Information.
