# Supplementary material for: Data-driven distillation and precision prognosis in traumatic brain injury with interpretable machine learning
Source: Sci Rep. 2023 Dec 1;13:21200. doi: 10.1038/s41598-023-48054-z (PMC10692236; doi:10.1038/s41598-023-48054-z)
Supplement: Supplementary file 1 — Supplementary Information. [file 41598_2023_48054_MOESM1_ESM.docx]

**Supplementary Material**

**
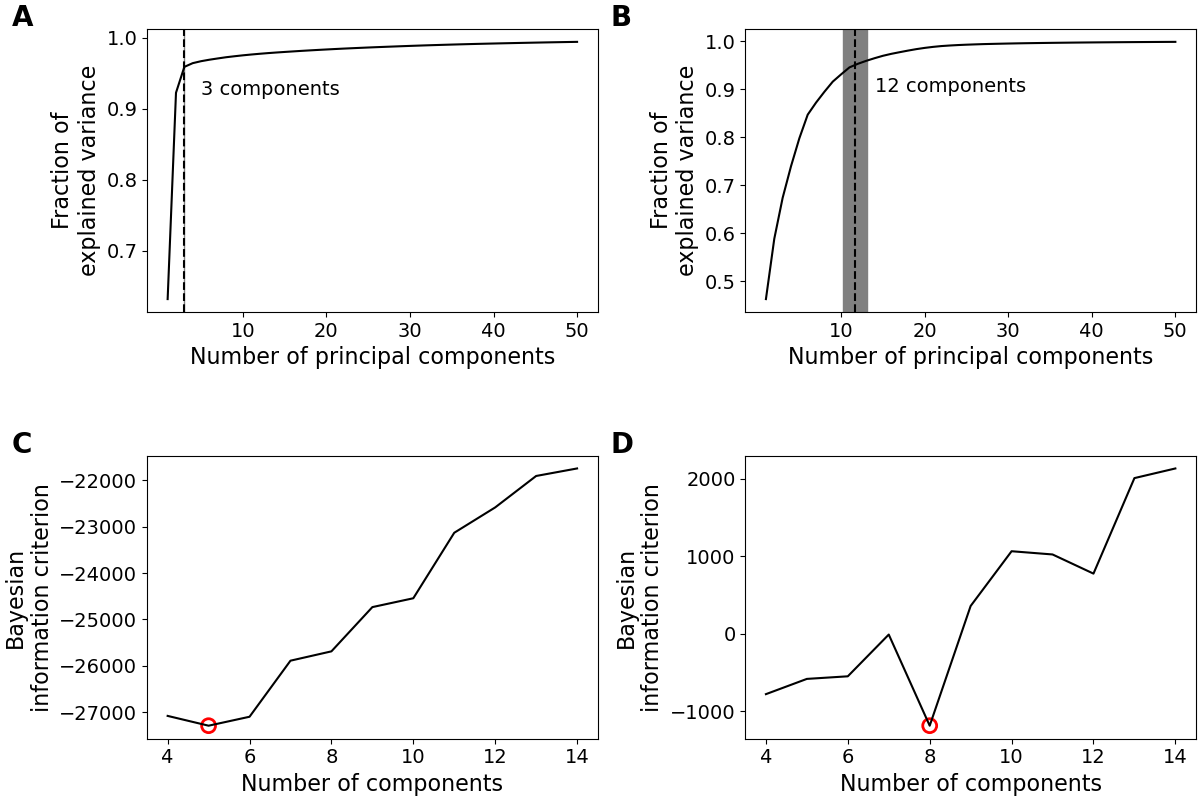
**

**Supplementary Figure 1. Dimensionality of PCA and NMF decompositions**

Number of latent features in intake feature and outcome features. **A,B**. Fraction of explained variance for principal components of all intake features (**A**) and all outcome features (**B**) across all patients (solid black lines). Vertical dotted line indicates the number of principal components required to capture 95% of explained variance, and shading around represents 95% confidence intervals over 1000 non-parametric bootstrap replicates. **C,D**. Bayesian information criterion (BIC) for the number of nonnegative factors in intake features (**C**) and outcome features (**D**) for all patients (solid black lines). Red circle indicates the number of components chosen (i.e. lowest BIC) for downstream analyses. Vertical dashed line and shading represent mean and 95% confidence intervals for the number of components estimated using the BIC across 1000 non-parametric bootstrap replicates. This revealed that the original selected number of latent dimensions the entire data set at or were at the average across bootstrap samples. For NMF on the outcome features, the mean across bootstraps was closer to 6. However, as our goal was to utilize bases that reconstruct data across all patients, we elected to retain 8 latent dimensions in the analysis of all patients (solid black line), which is within the 95% confidence intervals of the bootstrap samples.

**
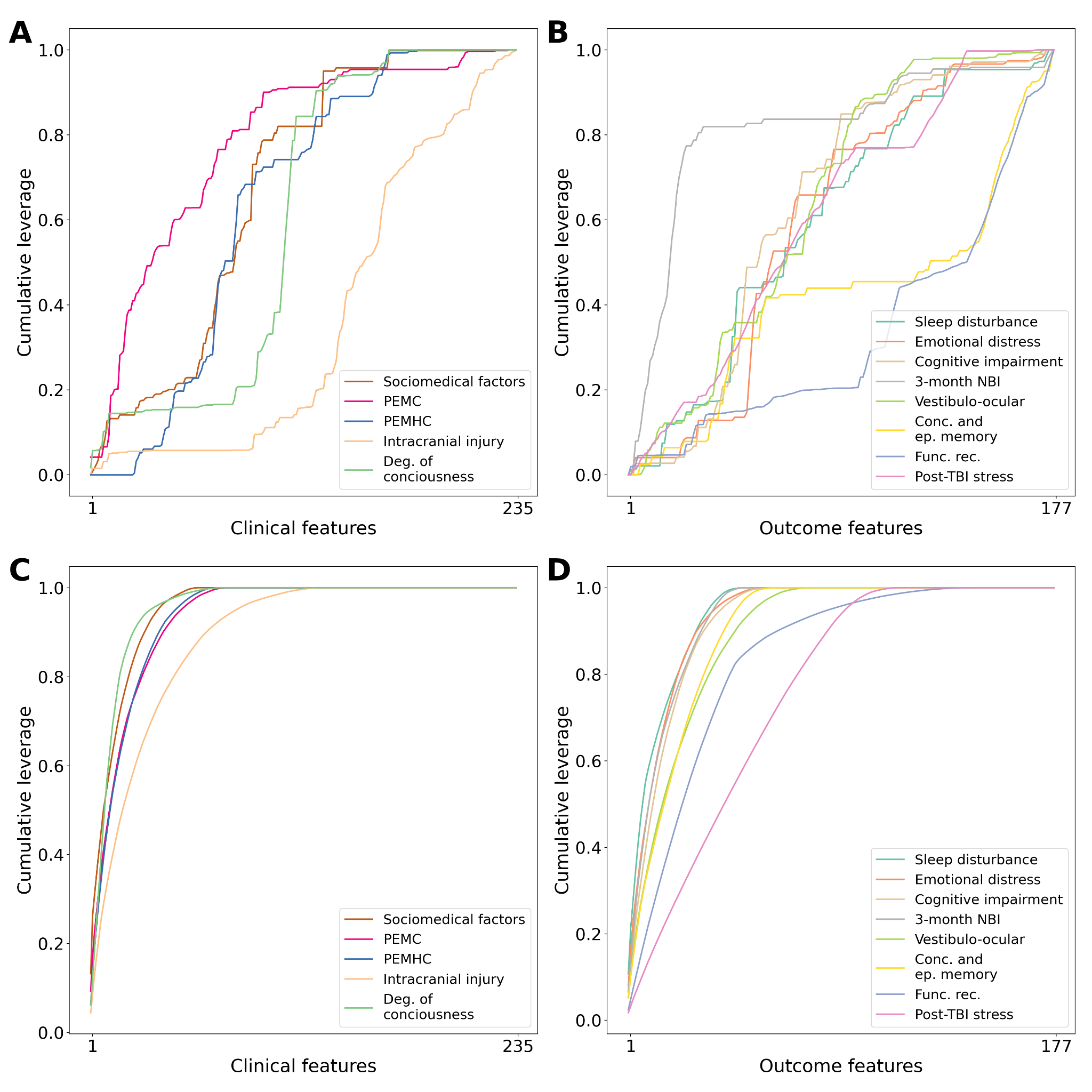
**

**Supplementary Figure 2. Feature leverage across nonnegative factors**

Cumulative normalized leverage of observed features across nonnegative latent factors. **A,B**. Arbitrary ordering of observed features for intake features (**A**) and outcome features (**B**). Ordering is consistent across features. **C,D**. Observed features sorted by leverage within each factor for intake features (**C**) and outcome features (**D**). Ordering is different for each factor.


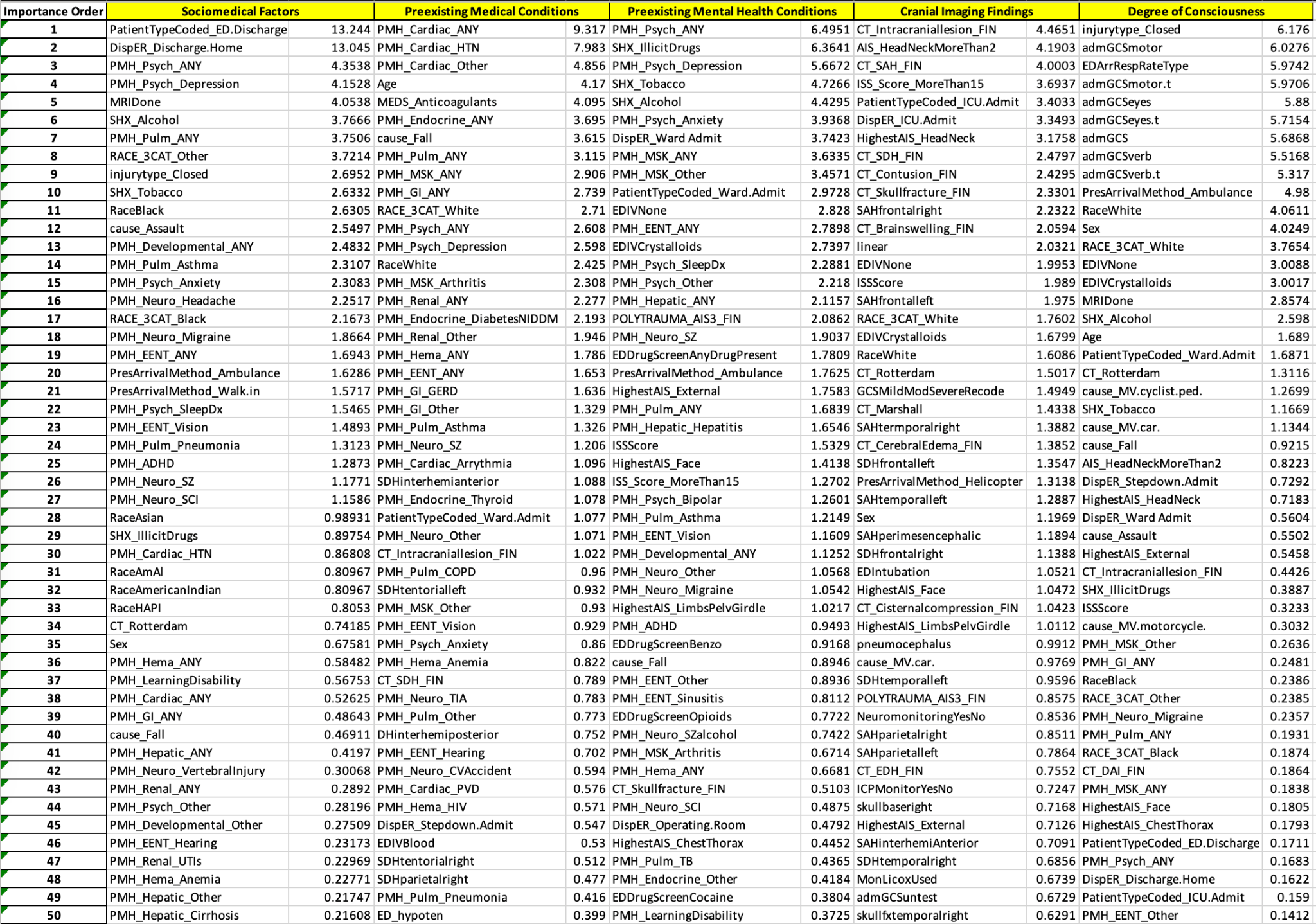
**Supplementary Figure 3. Feature leverage across nonnegative factors for intake variables.** Here we present an expanded view of the raw intake variable names and loadings for the intake NMF factors. Review of these data was used to name the factors.

**Supplementary Figure 4. Feature leverage across nonnegative factors for outcome variables.** Here we present an expanded view of the raw outcome variable names and loadings for the outcome NMF factors. Review of these data was used to name the factors. Continued on next page.


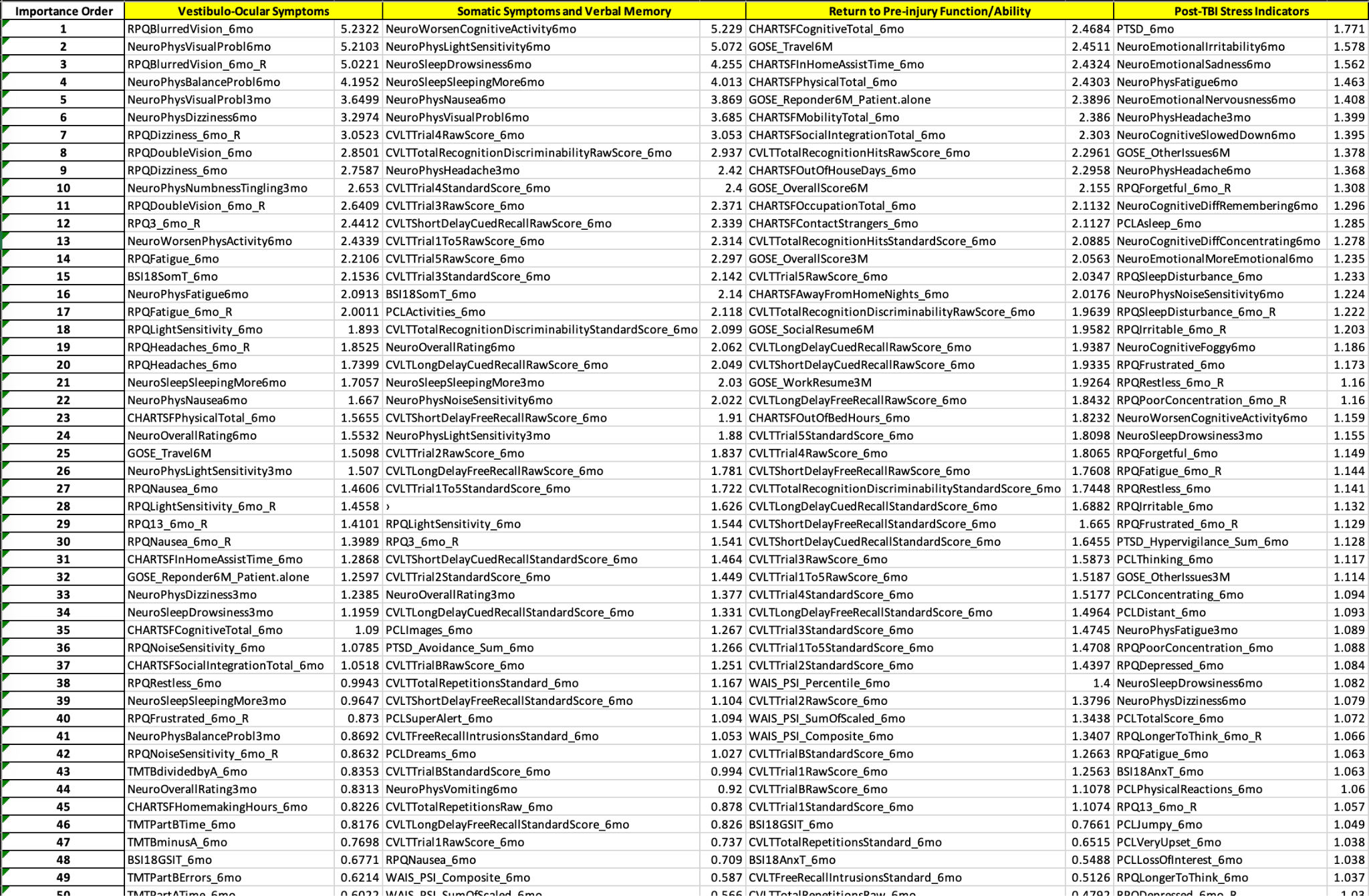


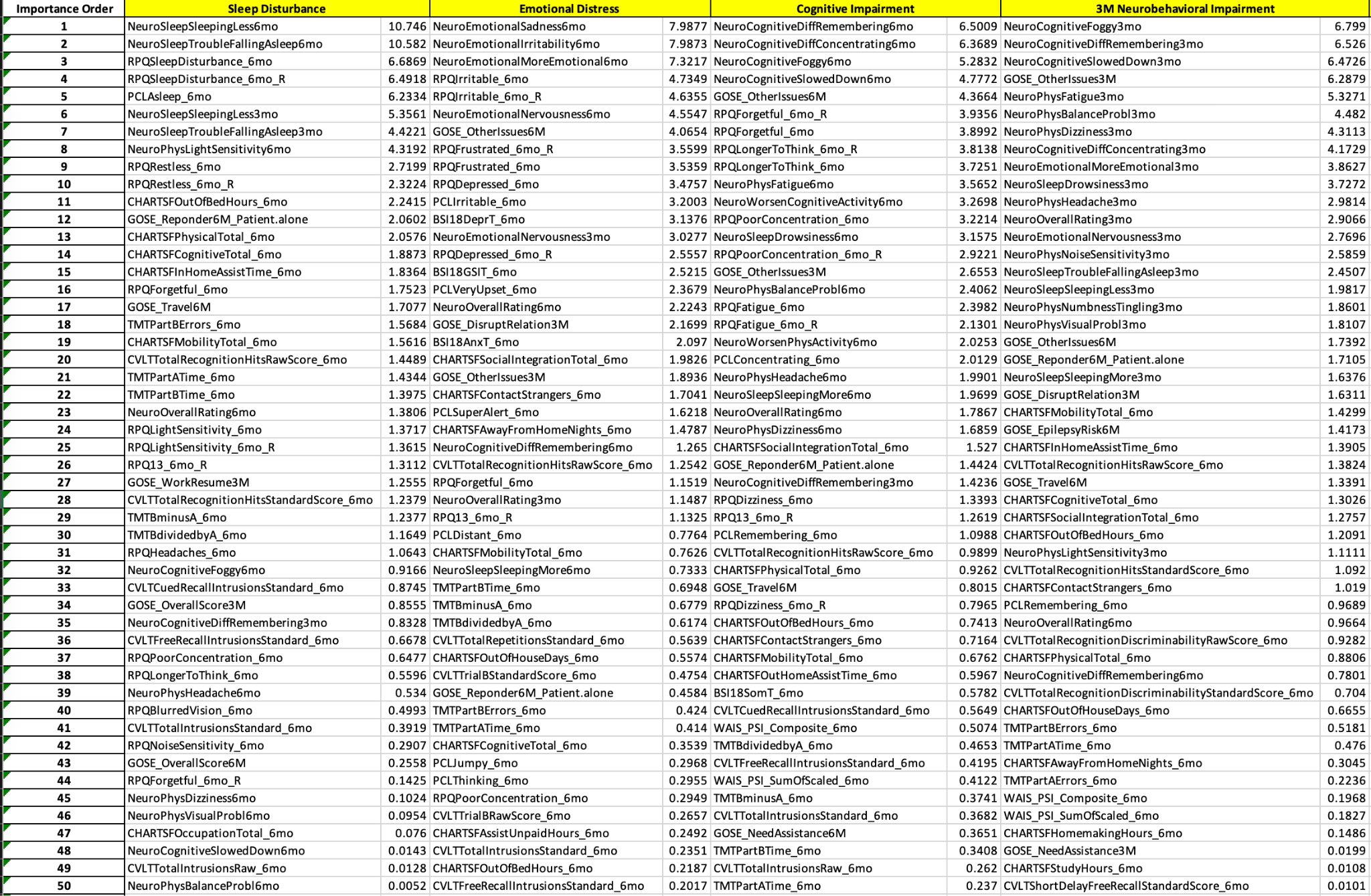


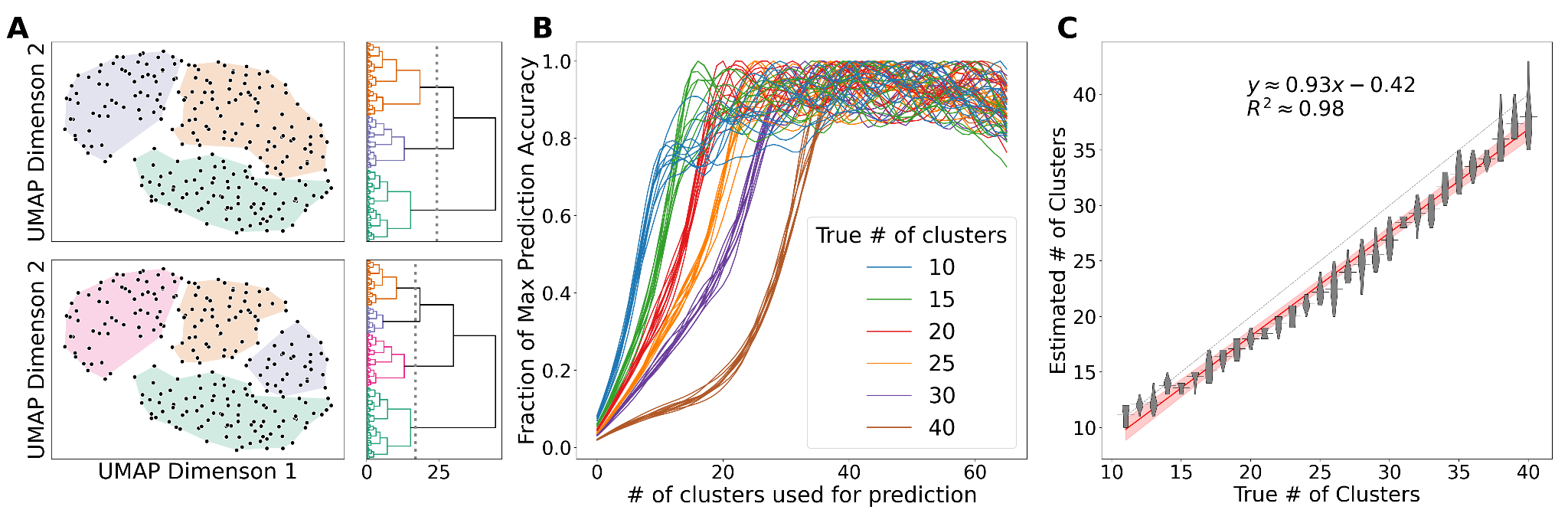
**Supplementary Figure 5. Estimating the number of outcome clusters.**

**A.** Clustering of UMAP outcome embeddings with hierarchical clustering. Top panel demonstrates cutting the hierarchical clustering dendrogram to have three clusters, and the bottom panel demonstrates cutting the hierarchical clustering dendrogram to have four clusters. **B.** Fold-over-chance predictive accuracy for different numbers of clusters in simulation data. Data was simulated using 2-50 clusters (some values were omitted for visualization purposes) for ten replicates. Each line corresponds to an individual replicate. Line colors correspond to the number of simulated clusters. Fold-over-chance predictive accuracy was calculated for 2-70 clusters by cutting hierarchical clustering dendrograms at different levels. **C**. Estimated number of clusters as a function of true number of clusters. Violin plots were calculated across replicates. Gray dotted line represents *y=x*. Red line represents the best fit line for regressing the estimated number of clusters onto the true number of clusters and the red shade represents the standard error of the estimates.

Here we describe our method for determining the number of outcome clusters and present results from simulated data. First, patients were clustered into different groups based on their outcome features using hierarchical clustering. We varied the number of outcome clusters that patients were grouped into by varying the threshold at which the resulting hierarchical clustering dendrogram is cut. For example, **SFig. 3A** shows the results for cutting the dendrogram to produce 3 (top) and 4 (bottom) clusters. Second, we trained a classifier to predict patient membership in the outcome clusters from the intake features and calculated cross-validated predictive accuracy. Note that the classification problem gets harder as the number of classes (i.e., outcome clusters) increases – that is, classification accuracy will generally decline as the number of classes increases. Thus, we normalized the predictive accuracy of the classifier by the data-derived chance accuracy, to arrive at the Fold Over Chance (FOC) metric. Third, we hypothesized that the true number of predictable clusters in the data is the smallest number of clusters for which the maximum FOC accuracy is achieved. Indeed, simulated data indicates this is true. In **SFigure 3B**, we plot FOC accuracy for simulated datasets as a function of the number of clusters used for prediction (see Methods). The different colors in **SFigure 3B** correspond to simulations with different numbers of ground-truth predictable clusters, and each line of a given color corresponds to a replicate of the synthetic data generation process. Here, we see that as the true number of clusters increased, so did the number of clusters at which the FOC asymptotes (x-axis). We estimated the point at which the FOC asymptote is first achieved by fitting the curves with a function that asymptotes (see Methods). We found that this approach was highly accurate at estimating the true number of predictable clusters in the synthetic data. In **Supplementary Figure 3C** we plot the distribution of the estimated number of clusters (y-axis) from our algorithm as a function of the true number of clusters (x-axis) in the simulated data (gray violins, dashed black line is unity). The redline corresponds to the best linear fit, which accounted for 98% of the variance in the estimated number of clusters (R^2^ = 0.98). We note that the slope of this line is slightly less than 1, and the offset is non-zero, indicating slight bias of the method. Nonetheless, we can use the best fit line to de-bias and quantify uncertainty on the estimated number of predictable clusters. Thus, we have developed a non-parametric, data-driven approach to determine the number of outcome clusters that can be predicted from the intake features.


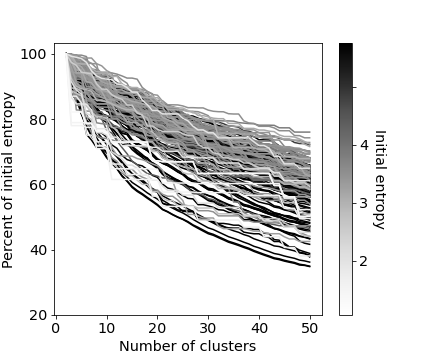


**Supplementary Figure 6. Entropy of outcome features as a function of number of outcome clusters**

Weighted mean entropy of outcome features as a function of number of outcome clusters. For each feature, weighted mean entropy across different clusterings is normalized by the entropy of the feature across the entire sample. Entropy for each feature at each clustering is calculated by taking the weighted mean entropy across all clusters, where mean entropy is weighted by the number of patients in each cluster.

**
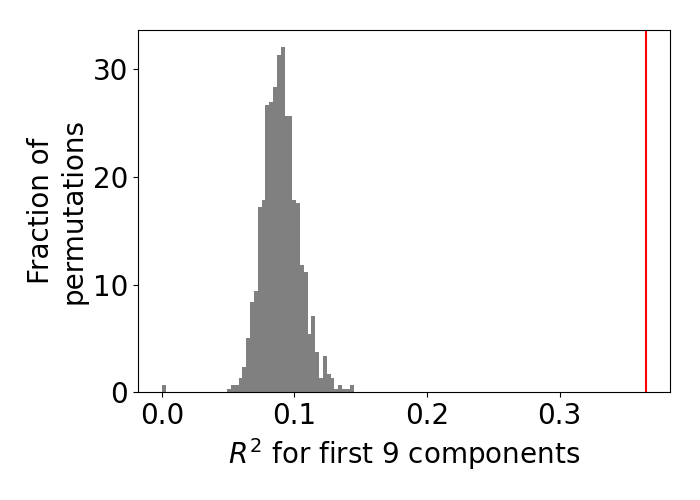
**

**Supplementary Figure 7. Permutation test results for CCA *R^2^***

Histogram of *R^2^* for first nine canonical variates calculated from 1024 permutations used in permutation test. Red line is the *R^2^* of the first nine canonical variates calculated from the original, un-permuted data (R^2^ = 0.36). The null distribution had a mean of 0.09 and a standard deviation of 0.014.

**
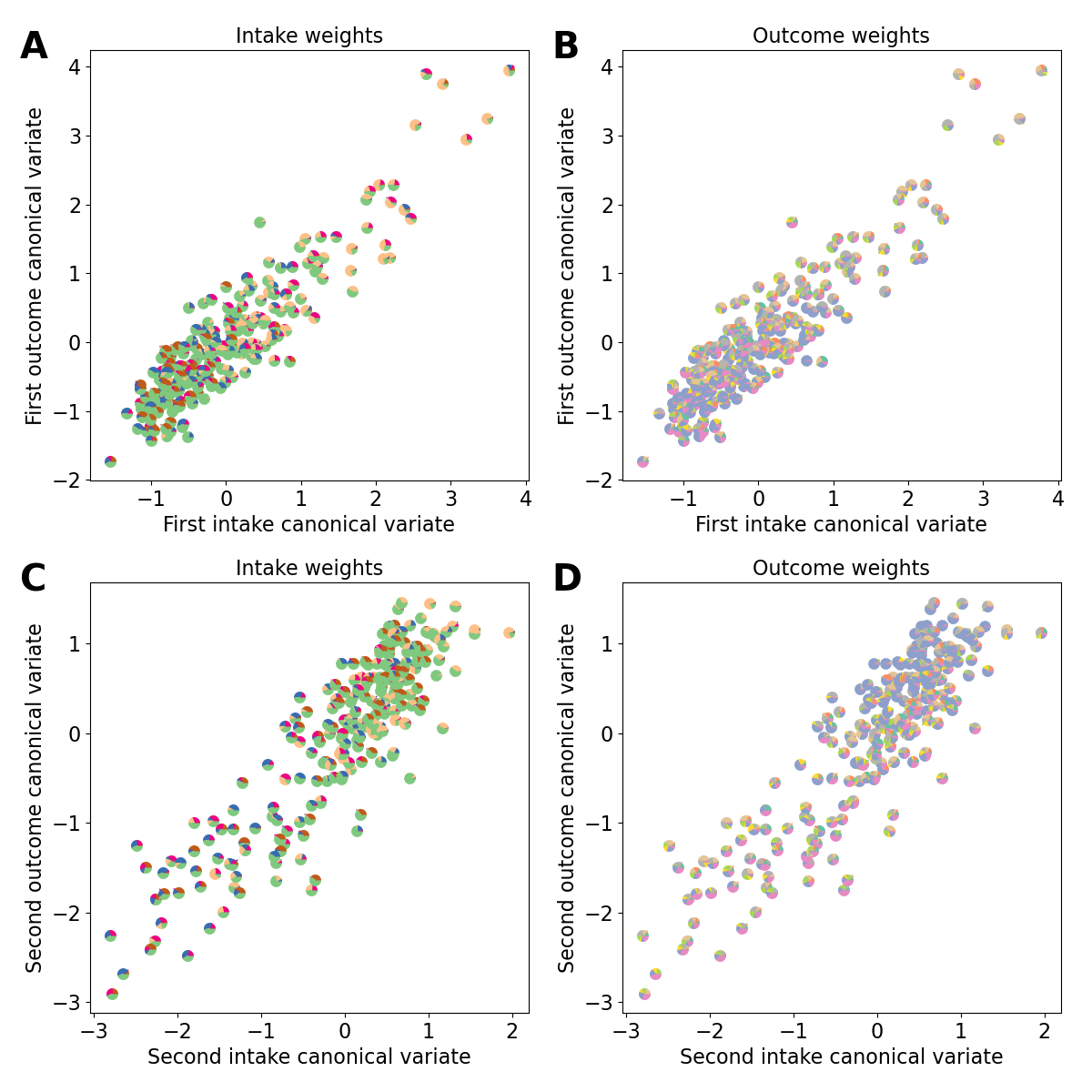
**

**Supplementary Figure 8. Visualization of nonnegative factors in CCA**

Canonical variates plotted as pie charts calculated using normalized NMF weights. **A**. First canonical variate plotted using intake nonnegative factors. **B**. First canonical variate plotted using outcome nonnegative factors. **C**. Second canonical variate plotted using intake nonnegative factors. **D**. Second canonical variate plotted using outcome nonnegative factors.

**
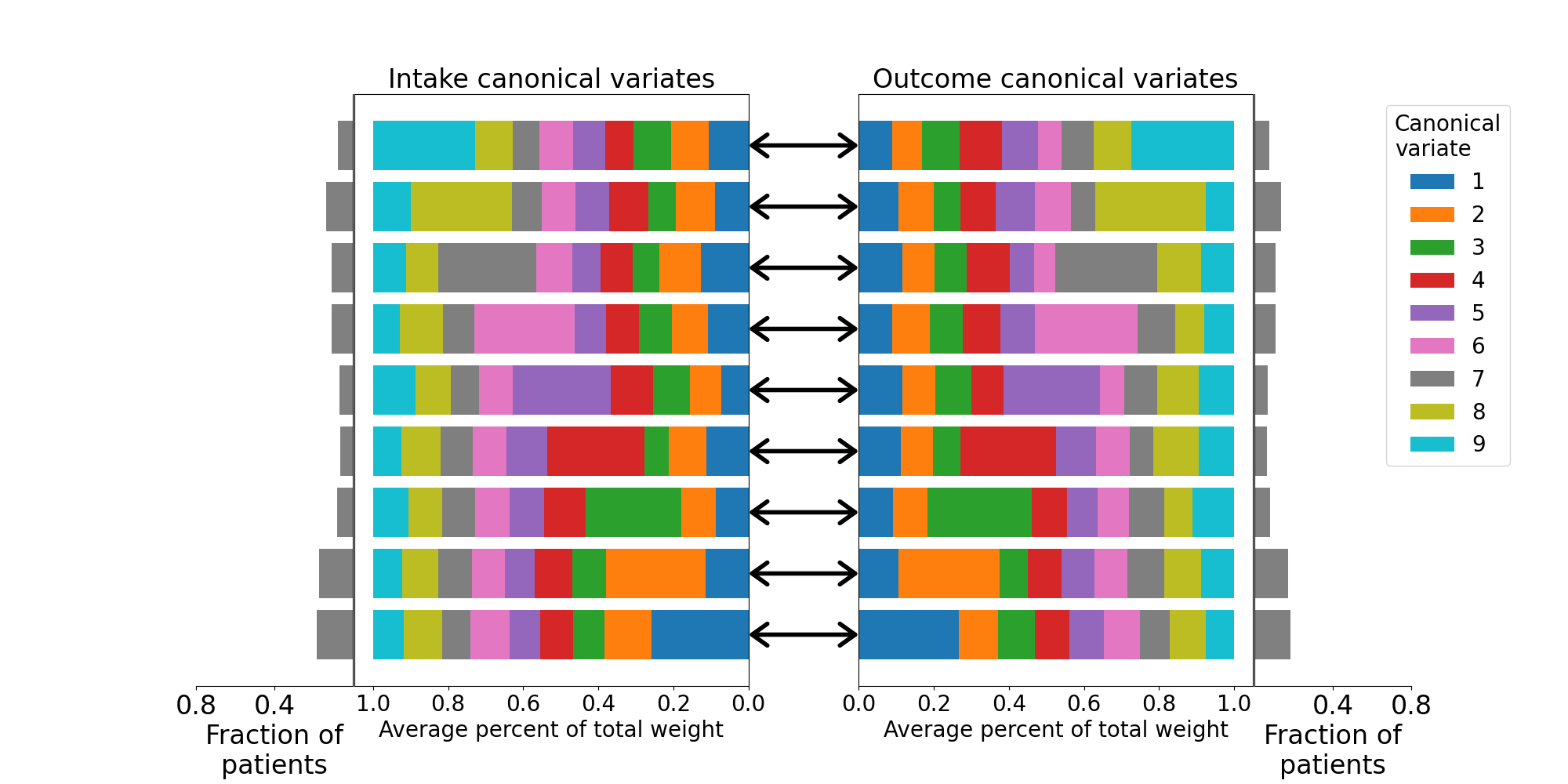
**

**Supplementary Figure 9. Visualization of CCA weightings across subjects** As in **Figure 3C,D**, we summarized the dominance and co-occurrence of patient canonical variates across the population. We first grouped patients according to their most heavily weighted canonical variate (9 CVs used). For each CV, we then averaged the CV contributions to the patients to create a summary representation. Note that CVs are paired across intake and outcome variables (demarcated by arrows in the figure). The outside panels display the fraction of patients that were dominant for each CV; inside panels display stacked bar plots of the distribution of CV contributions across patients dominant for that CV.

**Supplementary Discussion**

When dissected into their component parts, the GCS and GOSE are descriptions of a latent clinical state for injury and for recovery respectively. Each individual component score measures a component of a clinical state. Uol-NMF enables the deconstruction of aggregate clinical measures that describe a more “general” state, in order to re-construct more “precise” phenotypes of injury. NMF provides the much-needed solution to the source of the historical challenges for injury classification and outcome prediction (data heterogeneity; imprecise TBI classification tools; inadequate accounting of collinearity). When latent phenotypes were distilled from the GCS components and the other presentation/injury features, the degree of consciousness phenotype was composed of multiple GCS components, which underscores the inherent utility of the GCS for clinical assessment. However, the GCS eyes and motor score, and the overall GCS, were top contributors to multiple intake phenotypes **(Fig. 2C)**, showing that assessment of GCS is relevant to multiple subsets of potentially distinct patients. Similarly, the ability for local travel, and need for in-home assistance, which are assessed by the GOSE and other functional measures, were high contributors to the functional recovery outcome domain, underscoring their importance to that domain; however, these features were also enriched across several other outcome domains (vestibulo-ocular; 3-month NBI; sleep disturbance) **(Fig. 2D)** to show that these questions compose multiple latent outcome domains and have priority when determining variables of interest for assessing TBI outcome.

Of additional clinical interest are outcome symptoms that are pervasive but were not dominant outcome phenotype factors. For example, headache is among the most common residual symptoms after TBI[^50^](https://paperpile.com/c/vcLjnR/blqH), and headache features were present in the outcome feature set. However, headache did not, in-and-of-itself, emerge as a dominant, holistic set of outcome phenotypes. Indeed, careful examination of feature loadings across outcome factors **(Supplementary Figure 4)** shows that headache features are in the top 50 most heavily weighted outcome features across many outcome phenotypes: Post-TBI Stress, Sleep Disturbance, Cognitive Impairments, Vestibular Ocular Symptoms, and Somatic Symptom and Verbal Memory. This confirms that headache symptoms are common across many outcome phenotypes after TBI.

TBI patients present very heterogeneously across their sociodemographics, medical histories, injury subtypes and severities, and institutional treatment modalities. Moreover, the breadth of outcome domains after TBI are numerous, and factors that predict subtypes of outcome may not be conserved across subtypes of patients, their comorbidities, and/or their injuries. This phenomenon has long hampered the ability of clinicians and researchers to accurately prognosticate outcome using factors at presentation and acute care to properly guide management. The derived phenotypes in our study encompass both underlying intake and outcome features. The phenotypes are composed of “clusters” of demographic, socioeconomic, injury-related and acute treatment-related factors (for intake features) and functional, cognitive, psychiatric, and post-concussive symptoms (for outcome features) that respectively present together with varying “weights”. The associations between these aggregate intake and outcome phenotypes constitute a novel, objective, and data-driven method as an initial step and proof-of-concept toward more precise prognostication of outcomes after TBI beyond traditional methods of prediction such as multivariate regression or dimension reduction. We have shown that the intake and outcome features distilled using our methods have holistic relevance to determining latent features that co-occur together into composite underlying phenotypes representative of larger subpopulations of TBI patients. Our goal is to further validate the methods used herein across larger and more heterogeneous prospective TBI datasets with similar heterogeneity and data granularity in presentation and outcomes. Better understanding of these intake and outcome phenotypes, and their relationships (e.g., described in Figure 5) across other TBI studies (e.g., the 18-center U.S. TRACK-TBI Study, <https://tracktbi.ucsf.edu/>) will establish the utility of our novel methods to assist in the understanding of the subtypes of patients at increased or decreased risk of certain symptom clusters, wherein clinical treatment decisions can be better determined.
